# Psilocybin modulates social behaviour in male and female mice in a time-dependent manner

**DOI:** 10.1038/s41386-026-02450-x

**Published:** 2026-05-25

**Authors:** Sheida Shadani, Kaspar McCoy, Lina Ong, Erika Greaves, Kyna Conn, Zane B. Andrews, Claire J. Foldi

**Affiliations:** 1 Department of Physiology, Monash University, Clayton, VIC Australia; 2 Monash Biomedicine Discovery Institute, Clayton, VIC Australia

**Keywords:** Neuroscience, Physiology

## Abstract

With the resurgence of psychedelic research and growing evidence of their therapeutic potential, there is an urgent need to understand how these compounds act across biological sexes. Despite widespread interest in their use for conditions marked by social impairments, including depression, anxiety, and anorexia nervosa, the influence of sex as a biological variable on the prosocial effects of psychedelics remains poorly understood. Indeed, enhanced connectedness, sociability and empathy are common outcomes of psychedelic use and these have shaped human social structures for millennia. Here, we investigated the sex-specific effects of a single dose of psilocybin (1.5 mg/kg) in C57BL/6 J mice across multiple aspects of social behaviour. Psilocybin acutely enhanced huddling and induced hypothermia selectively in female mice and post-acutely (4 h) enhanced novelty-seeking and grooming in females, with no comparable effects in males. By 24 h, psilocybin-treated males showed reduced grooming and rearing alongside increased sociability directed toward a cage-mate. This was accompanied by blunted novelty-evoked nucleus accumbens dopamine responses that persisted to 7 days post-administration. At 7 days, psilocybin shifted female social preference toward familiarity over novelty, associated with prolonged nucleus accumbens dopamine release during familiar conspecific interactions, while males exhibited increased grooming, opposing the effect observed at 24 h. Both 5-HT1AR and 5-HT2AR contributed to psilocybin’s behavioural effects in sex-specific ways. These findings reveal temporally dynamic, sex-differentiated patterns of social behaviour and dopaminergic modulation following psilocybin, underscoring the importance of sex-informed approaches in preclinical research and clinical application of psychedelic compounds.

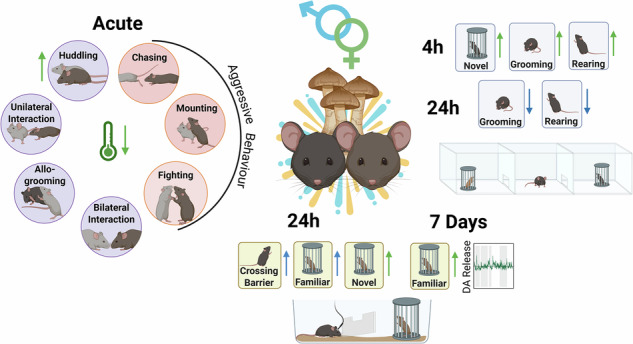

## Introduction

Social behaviour is fundamental to human cognition, shaping societal networks through cooperation, affiliation and trust [1]. The ability to form and maintain social bonds is crucial for psychological well-being and social deficits characterise numerous psychiatric and neurological disorders, including autism spectrum disorder, schizophrenia, depression and anorexia nervosa [2, 3]. Emerging evidence suggests that psychedelics, particularly psilocybin, may enhance social cognition and promote prosocial behaviour by modulating neural circuits involved in social reward and emotional processing [4, 5].

The resurgence of psychedelic research has revealed therapeutic potential for major depressive disorder, generalised anxiety disorder, obsessive-compulsive disorder, post-traumatic stress disorder (PTSD) and anorexia nervosa [6–14]. However, despite the central role of social behaviour in mental health, the impact of psychedelics on social cognition and motivation remains relatively underexplored. Current research has focused on primary symptom reduction in clinical populations, overlooking the potential for psychedelics to enhance social functioning that may be critical for long-term therapeutic efficacy. Expanding research into the social effects of psychedelics could offer novel mechanistic insights and refine clinical applications in disorders characterized by social impairments.

Emerging evidence suggests that psychedelics such as lysergic acid diethylamide (LSD) and psilocybin can enhance social connectivity in humans by modulating affective processing and social cognition [15]. Psilocybin has been shown to improve recognition of negative emotional stimuli [16], reduce feelings of social exclusion [17] and enhance empathy and prosocial behaviours [18], though these effects are not consistently observed across all studies [19]. These effects have been observed in both healthy individuals and patients with major depressive disorder [16, 17, 20, 21], supporting a role for psilocybin in fostering interpersonal connectedness [22]. Furthermore, single doses of psilocybin produce long-lasting improvements in social functioning and mood [23–25], indicative of persistent neuroplastic adaptations that enhance social engagement [26, 27]. Healthy volunteers also report elevated positive mood and prosocial behaviours under acute psilocybin effects [4, 28, 29], reinforcing its potential as a modulator of affective and social processing.

Preclinical studies investigating the influence of psychedelics on social behaviour in rodents show mixed outcomes. Psilocybin elicits both prosocial and antidepressant-like effects in male rats [30], while LSD and psilocybin both increase social interaction, preference for social novelty [31–34] and social reward learning [35] in male mice. However, a recent multi-institutional study failed to replicate effects of psilocybin on social reward learning or preference for novel social stimuli in male and female mice [36]. Notably, neither study reported or discussed potential sex-specific differences in sociability, underscoring the need for standardised protocols, reproducibility efforts, and separate analysis of both sexes to evaluate sex-dependent effects.

While interest in the behavioural effects of psychedelics has increased, underlying neurobiological mechanisms remain inadequately understood. The medial prefrontal cortex (mPFC), crucial for social decision-making and regulation of prosocial behaviours [37–39], is the most commonly reported site of psychedelic effects in human imaging and animal studies. However, psilocybin also modulates activity within the anterior cingulate cortex (ACC), involved in social pain processing [17], helping behaviour and empathy [40, 41] and social decision making [42], as well as the amygdala, which regulates social anxiety and threat perception [43]. These regional effects are linked to interactions with the mesolimbic dopamine system and the oxytocin-producing pathways, both of which are known to regulate pair bonding [44]. For instance, MDMA reopens social reward learning windows via oxytocin receptor activation in the nucleus accumbens [45], while LSD and psilocybin modulate connectivity between the default mode network (DMN) and social processing hubs, potentially reducing egocentric bias and increasing social connectedness [32]. Rodent studies further implicate the paraventricular nucleus of the hypothalamus (PVN), a key oxytocin-producing site, as a target for psychedelic-induced social facilitation [46]. Collectively, these findings suggest that psychedelics reshape social behaviour by engaging cortical and subcortical circuits that regulate social motivation, reward and emotional salience.

Despite promising findings, the majority of psychedelic research in humans and animal models has disproportionately (or often times exclusively) focused on male subjects, hindering generalizability given well-documented sex differences in neurobiology, psychiatric disease prevalence, and pharmacological responses [47, 48]. For instance, selective serotonin reuptake inhibitors (SSRIs), which target the serotonergic system key to psychedelic actions, exhibit sex-dependent efficacy and side-effect profiles [49]. Moreover, conditions such as depression, anxiety, PTSD and eating disorders, many being potential targets for psychedelic therapy, are significantly more prevalent in females [50–52]. To address this critical gap and align with the National Institutes of Health mandate to integrate sex as a biological variable (SABV) in experimental design [53], the present study investigated the effects of psilocybin on multiple aspects of social behaviour in both male and female mice and across different time points (from acute to post-acute administration). In addition, we tested the hypothesis that psilocybin binding to specific serotonin (5-HT) receptor subtypes is necessary for eliciting prosocial behaviour and paired social behaviour tasks with in vivo fiber photometry to investigate the neuronal substrates underlying these effects.

## Methods

### Animals and housing

Male (*n* = 182) and female (*n* = 145) C57Bl/6 mice (10 weeks old) were obtained from the Monash Animal Research Platform and pair-housed under reverse light cycle conditions (lights off at 0800 h). Mice acclimated for 7 days before testing, which occurred between 1000 h and 1700 h. Each mouse underwent only one experimental condition. All experimental procedures were approved by the Monash Animal Research Platform Ethics Committee (ERM 30852, ERM 40619).

### Drug administration

Psilocybin (PSI; 1.5 mg/kg) was administered intraperitoneally in saline. In receptor antagonist experiments, MDL100907 (5-HT2A receptor antagonist; 0.1 mg/kg) or WAY100635 (5-HT1A receptor antagonist; 0.5 mg/kg) were administered 30 mins prior to PSI or saline (SAL) control. Doses were selected based on previous studies [54]. See **Supplementary Methods** for drug preparation and supplier details.

### Behavioural testing

#### Home-cage social behavior

Following PSI or SAL injection, pair-housed mice were video-recorded for 1 h in their home cage. Social behaviours, including bilateral (reciprocal interactions involving active engagement from both mice) and unilateral interactions (initiated by one mouse without a corresponding response), as well as huddling, allogrooming, mounting and chasing, were manually scored using Ethovision XT software.

#### 3-Chamber test

Mice were tested at 4 h, 24 h or 7 d post-injection using the standard 3-chamber paradigm. Two strain-, age- and sex-matched novel mice were used as social stimuli. Following 10 min habituation, mice explored a novel mouse versus empty cage (social preference trial), then a second novel mouse versus the now-familiar mouse (social novelty trial). Time in chambers, interaction frequency and locomotor activity were quantified. Social preference was calculated as (T_s_ - T_ns_)/ (T_s_ + T_ns_), where T_s_ is time spent with the novel mouse and T_ns_ is time spent with the empty cage or familiar mouse. Sociability Z-scores were calculated to assess changes in sociability across treatment groups relative to saline-treated (control) animals by normalizing individual scores to control group means: Z = (x - μ)/σ, based on chamber duration and entry frequency [55].

#### Barrier climbing test

Mice were assessed for social motivation using an adapted maternal motivation paradigm [56–58]. This was included as an alternative measure of social behaviour that is compatible with concurrent fiber photometry recordings. Following 10 min habituation to a 60 mm transparent barrier, mice explored a novel mouse (5 min) then a cage mate (5 min), each confined in a wire cage on the opposite side of the barrier. Latency to first climb, total climbs and latency to sniff were recorded.

#### Acute physiological monitoring

Core body temperature and locomotor activity were continuously monitored using subcutaneously implanted RFID microchips (UCT-2112; Unified Information Devices) in the home cage for 1 h before and 2 h after injection.

### Fiber photometry

Mice received unilateral injections of hSyn-GRAB_DA2m_ (Addgene #140553) and optical fiber implantation (AP: + 1.4, ML: ±0.75, DV:-4.2 mm). Following 5 weeks of recovery, dopamine dynamics were recorded during the barrier climbing task using 470 nm (signal) and 410 nm (isosbestic control) excitation within the RWD R821 system. See **Supplementary Methods** for extended surgical and viral injection procedure details.

### Statistical analyses

Data were analysed using GraphPad Prism 9.5.1 with significance set at *p* < 0.05. Analyses included unpaired t-tests, one-way and two-way ANOVA with Sidak’s post hoc tests and mixed effects models as appropriate to evaluate sex differences in behavioural parameters and the effects of psilocybin within each sex. Complete statistical details are provided in the **Supplementary Materials**.

## Results

### Psilocybin acutely enhances social thermoregulation selectively in female mice

**Fig. 1:**
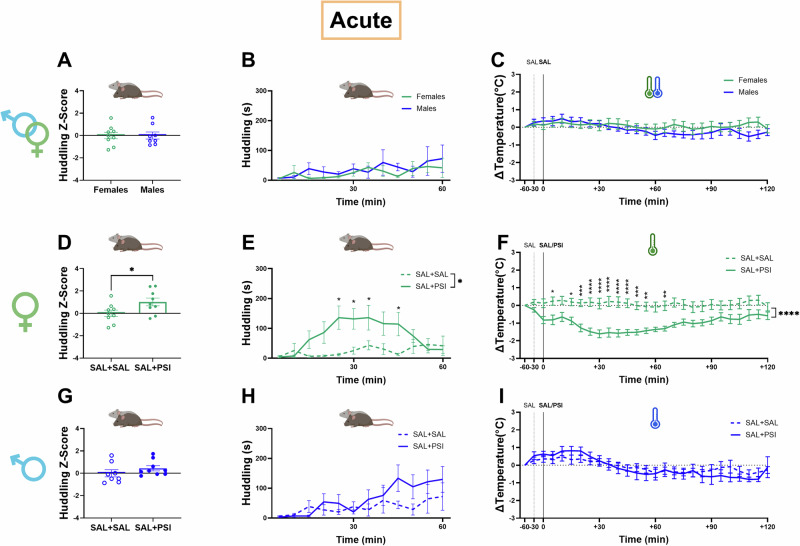
Female but not male mice show acute psilocybin-induced increases in huddling and reductions in core body temperature, with no baseline sex differences. **A, B** Male and female mice did not differ significantly in baseline huddling behaviour. **C** Baseline core body temperature fluctuated differently over time between male and female mice, as indicated by a significant time x sex interaction (*F* (5.541, 81.62) = 2.833, *p* = 0.0173). **D** In female mice, psilocybin significantly increased huddling behaviour compared to the controls (*p* = 0.0391) where **E** huddling peaked at 25 min (*p* = 0.0133), 30 min (*p* = 0.0155) and 45 min (*p* = 0.0311) post-administration. **F** Psilocybin significantly reduced core body temperature in a time-dependent manner in female mice between 5-70 min post-administration (*F* (1160) = 16.1, *p* = 0.001). **G** In male mice, total huddling behaviour was not significantly affected by psilocybin, **H** no time-dependent differences in huddling behaviour were observed over 60 min and **I** core body temperature was not significantly altered by psilocybin. Psilocybin (PSI), saline (SAL). Female and male subjects are represented in green and blue, respectively. Saline-treated animals are represented by dashed lines and open symbols, while psilocybin-treated animals are represented by solid lines and filled symbols. Z-score = normalised measure relative to saline-treated animals. Data are presented as mean ± SEM. Statistical analyses were performed using unpaired t-test, mixed-effects analysis or two-way ANOVA with Šidák post hoc tests. *p < 0.05, ****p < 0.0001. Schematics created in BioRender.

To examine whether male and female mice differed in baseline huddling behaviour and core body temperature, we assessed these measures in saline-treated mice and observed no significant differences in either huddling behaviour (Fig. 1A, B) or core body temperature (Fig. 1C), confirming that baseline sex differences do not confound subsequent comparisons. To evaluate the acute effects of psilocybin on home-cage social behaviour, we examined huddling in pair-housed mice. Psilocybin significantly increased huddling in females within 60 min post-administration (*p* = 0.0391; Fig. 1D), with peak effects at 25 min (*p* = 0.0133; Fig. 1E). This coincided with significant reductions in core body temperature beginning at 5 min (*p* = 0.0277) and persisting until 70 min (*p* = 0.0073; Fig. 1F). Psilocybin alone did not alter huddling behaviour (Fig. 1G, H) or core body temperature in male mice (Fig. 1I).

### Psilocybin-induced social thermoregulation in female mice is 5-HTR dependent and huddling behaviour in psilocybin-treated male mice is modulated by 5-HT2AR and 5-HT1AR antagonism

In female mice, 5-HT1AR antagonism abolished huddling regardless of treatment (saline: *p* = 0.0062; psilocybin: *p* < 0.0001), while 5-HT2AR antagonism selectively reduced huddling only in psilocybin-treated mice (*p* = 0.0029), demonstrating receptor-specific modulation of psilocybin’s acute social effects. Both antagonists reduced core body temperature in saline controls; however, in psilocybin-treated mice, 5-HT2AR antagonism increased temperature (*p* = 0.0398), remaining elevated between 60-90 min, whereas 5-HT1AR antagonism prolonged hypothermia. In male mice, both 5-HT2AR and 5-HT1AR antagonism significantly reduced huddling in psilocybin-treated but not saline-treated mice (*p* = 0.0018), revealing sex-differentiated receptor contributions to psilocybin’s behavioural effects (Supplementary Data 1). 5-HT2AR antagonism reduced unilateral interactions in females regardless of psilocybin treatment (*p* = 0.0339 saline; *p* = 0.0328 psilocybin), while both antagonists reduced aggressive behaviour (primarily mounting, followed by chasing, pinning and fighting) in saline-treated males, effects that were absent when combined with psilocybin (Supplementary Data 1). To isolate receptor-specific effects, we compared psilocybin versus saline within each antagonist pre-treatment group (Supplementary Data 2). In females, both 5-HT1AR and 5-HT2AR receptor antagonism abolished psilocybin-induced huddling while hypothermia persisted. In males, psilocybin produced opposite effects on huddling depending on which receptor was blocked, revealing sex-differentiated receptor mechanisms underlying psilocybin’s acute behavioural effects.

### Psilocybin modulates novelty-seeking behaviour post-acutely in female mice through 5-HT1AR and 5-HT2AR mechanisms

We used the 3-chamber test to assess how psilocybin impacts social preference and novelty-seeking behaviour. At 4 h post-administration, psilocybin enhanced novelty-seeking in female mice (preference for novel mouse, *p* = 0.0494) and increased grooming (*p* = 0.0443) without affecting locomotion. Both 5-HT2AR and 5-HT1AR antagonism attenuated psilocybin-induced sociability (*p* < 0.03); however, only 5-HT2AR blockade was specific to social behaviour, while 5-HT1AR antagonism also reduced locomotion, complicating interpretation. Neither antagonist affected sociability in saline-treated controls, confirming selective modulation of psilocybin’s effects. Male mice showed no psilocybin-induced changes in sociability or stereotypic behaviours at this timepoint (Supplementary Data 3–4).

### Psilocybin attenuates rearing and grooming in male mice at 24 h post-administration, but does not alter sociability

**Fig. 2:**
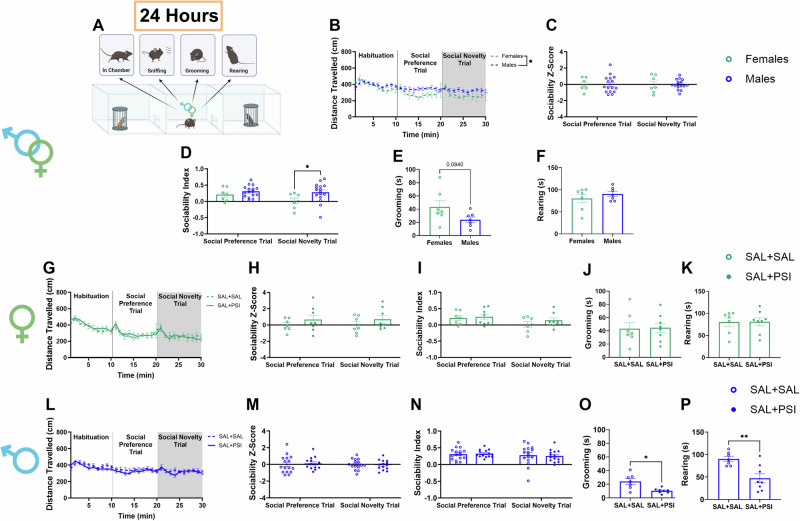
Psilocybin reduces grooming and rearing but not sociability in male mice 24 h after administration, despite higher baseline sociability compared to females. **A** Schematic of the social novelty trial and the behaviours scored. **B** Baseline locomotor activity showed sex-dependent variation over time, as indicated by a significant time x sex interaction (*F* (9.42, 197.8) = 2.217, *p* = 0.0206). **C** Sociability Z-score did not differ between males and females during the social preference or novelty trials; however, during the social novelty trial, **D** male mice exhibited a higher sociability index than females (*p* = 0.0341). **E** Female mice showed a non-significant trend towards higher grooming behaviour compared to males (*p* = 0.094) and **F** rearing behaviour was similarly comparable. **G** Psilocybin did not affect locomotor activity in female mice across the experiment. **H, I** Sociability was similarly unaffected, nor were (**J**) grooming or **K** rearing behaviours affected. In male mice, psilocybin also had no effect on **L** locomotor activity or **M, N** sociability scores. However, it significantly increased (**O**) grooming (*p* = 0.0159) and reduced **P** rearing behaviour (*p* = 0.0033). Psilocybin (PSI), saline (SAL). Female and male subjects are represented in green and blue, respectively. Saline-treated animals are represented by dashed lines and open symbols, while psilocybin-treated animals are represented by solid lines and filled symbols. Z-score = normalised measure relative to saline-treated animals; sociability index = relative direct interaction time between the social and non-social stimuli. Data are presented as mean ± SEM. Statistical analyses were performed using unpaired t-test or two-way ANOVA with Šidák post hoc tests. *p < 0.05, **p < 0.01. Schematics created in BioRender.

Given the possibility that psilocybin’s time course differs between sexes, we conducted the 3-chamber test in male and female mice at 24 h (Fig. 2A). We observed a significant time x sex interaction in locomotor activity (*F* (9.42,197.8) = 2.217, *p* = 0.0206; Fig. 2B). Male and female mice exhibit a comparable sociability Z-score (Fig. 2C); however, during direct interaction, male mice showed significantly higher sociability toward a novel mouse than females (*p* = 0.0341; Fig. 2D). Female mice showed a non-significant trend toward higher grooming behaviour compared to males (*p* = 0.094; Fig. 2E), while rearing behaviour was comparable between sexes (Fig. 2F). Psilocybin did not affect locomotor activity in female mice (Fig. 2G), nor did it affect sociability measures (Fig. 2H, I) or stereotypic behaviours (Fig. 2J, K). Similarly, in male mice, psilocybin did not affect locomotor activity (Fig. 2L) or sociability measures (Fig. 2M, N), but significantly reduced grooming (*p* = 0.0159; Fig. 2O) and rearing (*p* = 0.0033; Fig. 2P).

At 24 h post-administration, 5-HT2AR antagonism significantly reduced sociability Z-scores in saline-treated male mice (*p* = 0.0168), an effect not observed in psilocybin-treated males. This pattern was similarly reflected in (raw) sociability indices, where 5-HT2AR antagonism reduced scores in saline-treated but not psilocybin-treated males (*p* = 0.0077). Although neither 5-HT2AR nor 5-HT1AR antagonism significantly affected sociability in psilocybin-treated mice, 5-HT1AR antagonist pre-treatment showed a non-significant trend toward reduced sociability (*p* = 0.0584). Neither psilocybin, 5-HT1AR antagonism, nor 5-HT2AR antagonism affected preference for a novel mouse over a novel object in male mice (Supplementary Data 5).

### Psilocybin increases preference for familiarity in female mice and grooming behaviour in males at 7 days post-administration

**Fig. 3:**
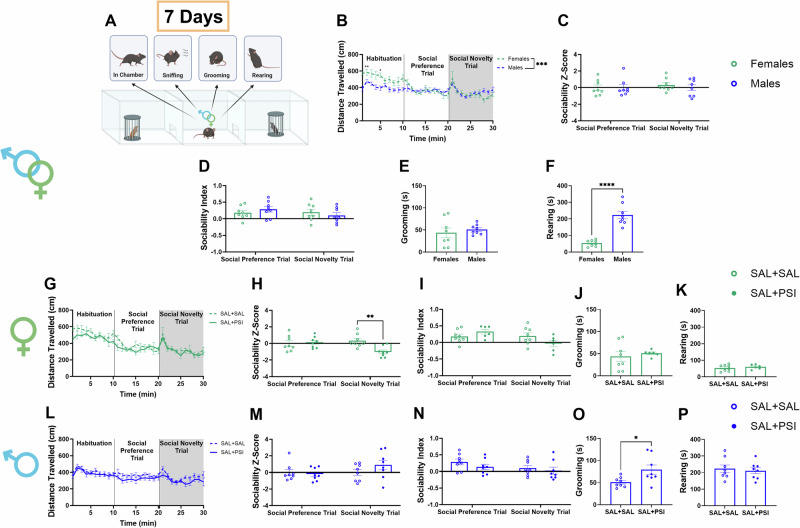
Psilocybin increased preference for familiarity in female mice and grooming behaviour in male mice 7 days post-administration. **A, B** A significant time x sex interaction was observed in locomotor activity (*F* (6.096,85.34) = 4.73, *p* = 0.0003) with females showing higher locomotor activity at 1 min (*p* = 0.0064) and 5 min (*p* = 0.0256) of the habituation trial. **C, D** Male and female mice did not differ significantly in baseline sociability measures, **E** nor did they differ in grooming behaviour. **F** However, male mice exhibited significantly higher rearing behaviour at baseline (*p* < 0.0001). **G** Psilocybin did not alter locomotor activity in female mice; **H** however, psilocybin significantly reduced sociability Z-score in these mice (*p* = 0.0023). **I** Psilocybin did not affect sociability index, **J** grooming or **K** rearing behaviours in female mice. **L** Psilocybin did not affect locomotor activity, **M** sociability Z-score or **N** sociability index in male mice. **O** Psilocybin increased grooming behaviour in male mice (*p* = 0.0273) and **P** had no effect on rearing behaviour. Psilocybin (PSI), saline (SAL). Female and male subjects are represented in green and blue, respectively. Saline-treated animals are represented by dashed lines and open symbols, while psilocybin-treated animals are represented by solid lines and filled symbols. Z-score = normalised measure relative to saline-treated animals; sociability index = relative direct interaction time between the social and non-social stimuli. Data are presented as mean ± SEM. Statistical analyses were performed using one-way ANOVA or two-way ANOVA with Šidák post hoc tests. *p < 0.05, **p < 0.01, ****p < 0.0001. Schematics created in BioRender.

To evaluate the effects of psilocybin at 7 d post-administration in the 3-chamber test (Fig. 3A), we compared baseline behaviours of male and female mice. We observed a significant time x sex interaction in locomotor activity (*F* (6.096,85.34) = 4.73, *p* = 0.0003), with female mice showing significantly higher locomotor activity than males during habituation at 1 min (*p* = 0.0064) and at 5 min (*p* = 0.0256; Fig. 3B). Male and female mice did not differ in sociability measures or grooming behaviour (Fig. 3C–E); however, males exhibited significantly higher rearing behaviour than females (*p* < 0.0001; Fig. 3F). At 7 d post-administration, psilocybin did not affect locomotor activity in female mice (Fig. 3G); however, it significantly reduced sociability Z-score only during the social novelty trial (*p* = 0.0031, Fig. 3H), suggesting a shift towards familiarity preference, without affecting other measures (Fig. 3I–K). In male mice, psilocybin did not affect locomotor activity (Fig. 3L) or sociability measures (Fig. 3M, N), but increased grooming behaviour 7 d following treatment (*p* = 0.0363; Fig. 3O), an effect opposing that observed at 24 h results, while rearing behaviour was unaffected (Fig. 3P).

### Psilocybin differentially modulates social motivation in male and female mice at 24 h

**Fig. 4:**
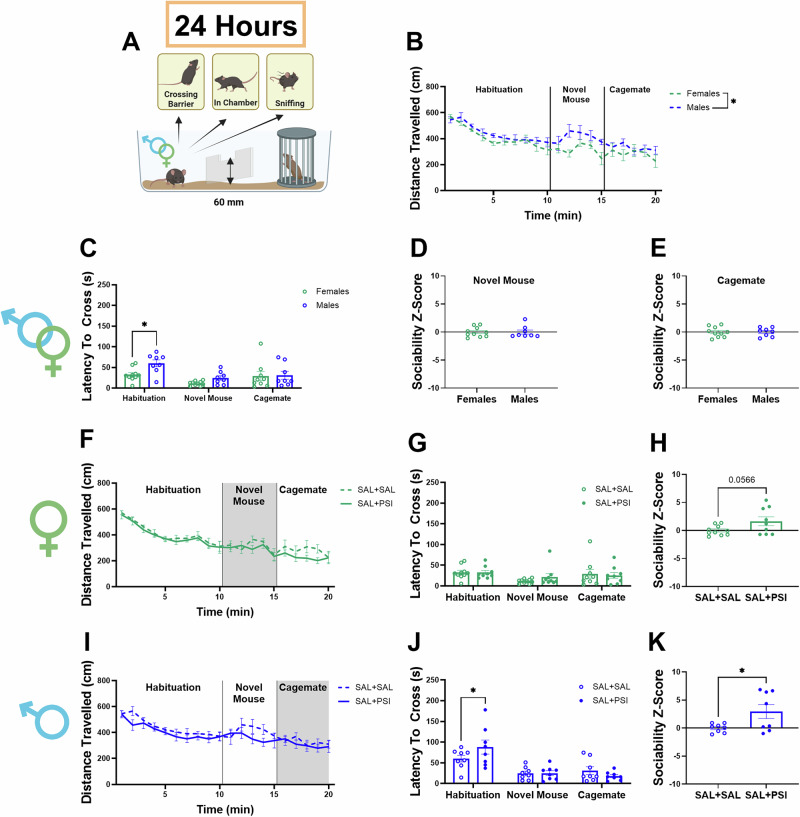
Male and female mice show comparable baseline sociability but divergent responses to psilocybin at 24 h. **A** Schematic representation of the barrier climbing test and the behavioural measures assessed. **B** Male and female mice differed significantly in baseline locomotor activity (*F* (1,16) = 5.992, *p* = 0.0263) and **C** male mice showed significantly higher latency to cross the barrier during habituation (*p* = 0.0443). Sociability Z-score of mice was comparable when exploring **D** the novel mouse or **E** their cage mates. **F** Locomotor activity and **G** latency to cross the barrier were unaffected by psilocybin in female mice. **H** Psilocybin-treated female mice showed a non-significant trend towards increased preference for novelty (*p* = 0.0566). **I** Psilocybin did not affect locomotor activity in male mice across the experiment. **J** Psilocybin increased latency to cross the barrier during habituation (*p* = 0.0407). **K** Psilocybin-treated male mice exhibited a significantly higher sociability Z-score toward their cage mates compared to the controls (*p* = 0.0349). Psilocybin (PSI), saline (SAL). Female and male subjects are represented in green and blue, respectively. Saline-treated animals are represented by dashed lines and open symbols, while psilocybin-treated animals are represented by solid lines and filled symbols. Z-score = normalised measure relative to saline-treated animals. Data are presented as mean ± SEM. Statistical analyses were performed using unpaired t-test, mixed-effects analysis or two-way ANOVA with Šidák post hoc tests. *p < 0.05. Schematics created in BioRender.

Since 3-chamber sociability is driven by motivational processes, we assessed the effects of psilocybin on social motivation using a barrier climbing test and observed distinct responses in males and females 24 h following administration of saline or psilocybin (Fig. 4A). Prior to evaluating the effects of psilocybin within each sex, we characterised baseline sex differences and identified a significant main effect of sex on locomotor activity, with males exhibiting higher activity than females (F (1,16) = 5.992, *p* = 0.0263; Fig. 4B). During habituation, male mice also showed longer latency to cross the barrier than females (*p* = 0.0443; Fig. 4C), while sociability was comparable between sexes when interacting with a novel mouse or (familiar) cage mate (Fig. 4D, E). In female mice, psilocybin did not alter locomotor activity (Fig. 4F) or latency to cross the barrier across different trial phases (Fig. 4G); however, psilocybin-treated females showed a non-significant trend toward increased social motivation, reflected in a higher sociability Z-score for a novel conspecific (*p* = 0.0566; Fig. 4H). In male mice, psilocybin had no effect on overall locomotor activity (Fig. 4I) but significantly increased the latency to cross the barrier upon arena introduction (*p* = 0.0407; Fig. 4J) and enhanced social preference toward cage mates (*p* = 0.0349; Fig. 4K).

When we evaluated the effects of 5-HT2AR and 5-HT1AR antagonism on these measures, latency to cross was unaffected in female mice regardless of treatment group. Psilocybin-elevated sociability showed a non-significant trend toward attenuation with 5-HT2AR but not 5-HT1AR antagonism (*p* = 0.0694). During cage mate exploration, neither 5-HT2AR nor 5-HT1AR antagonism, alone or in combination with psilocybin, affected locomotor activity or social preference in female mice (Supplementary Data 6). In male mice, neither 5-HT2AR nor 5-HT1AR antagonism affected locomotion, social motivation or sociability under saline or psilocybin-treated conditions, regardless of whether mice explored a familiar or novel conspecific (Supplementary Data 7).

### Psilocybin elicits differential alterations in nucleus accumbens (NAc) dopamine release in males versus females at 24 h and 7 days post-administration

**Fig. 5:**
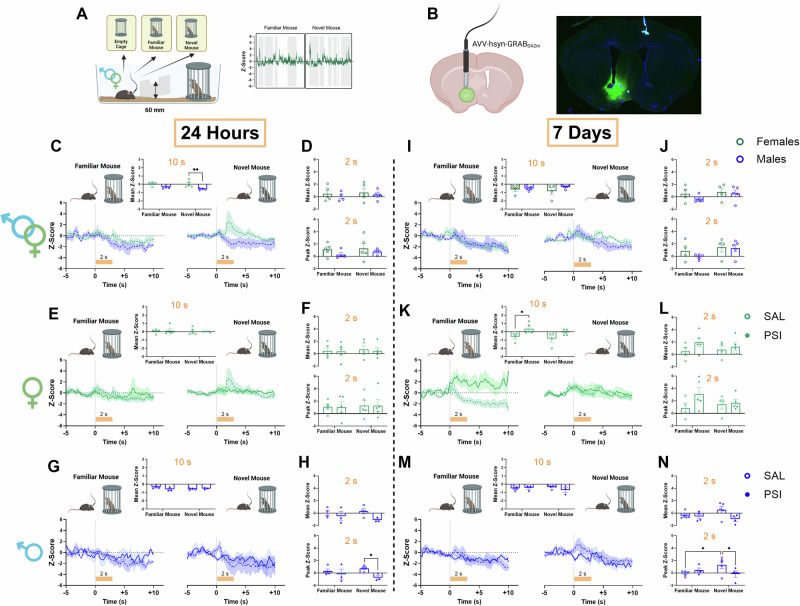
Male mice show lower baseline NAc dopamine (DA) than females in response to novelty and psilocybin differently modulates dopamine release in each sex across time. **A** Schematic of the experimental design and the average GRAB_DA2m_ signal across the full trial when the mouse was in the social zone (shaded area) versus the non-social zone. **B** Representative fiber placement and virus injection in the NAc. **C** Average GRAB_DA2m_ traces from the first interaction with a familiar or novel mouse at 24 h post-administration in male and female mice. Inset: Male mice had a significantly lower DA Z-score than female mice when interacting with a novel mouse during the first 10 seconds (*p* = 0.0082); **D** however, males and females did not differ in DA release or peak DA release during the first 2 seconds of interaction with a familiar or novel mouse. **E** Average GRAB_DA2m_ traces from the first interaction with a familiar or novel mouse at 7 days post-administration in male and female mice. Inset: No significant difference between males and females was observed in DA release when interacting with a familiar or novel mouse during the first 10 seconds. **F** Similarly, no significant difference in immediate DA release was observed between sexes. **G** Average GRAB_DA2m_ traces from the first interaction with a familiar or novel mouse at 24 h post-administration of psilocybin or saline in female mice. Inset: No significant effect of PSI on DA release was observed during the first interaction with either mouse. **H** At 24 h, psilocybin did not alter DA release within the first 2 seconds of sniffing either mouse. **I** At 7 days post-administration, average traces show prolonged DA release in response to familiar (but not novel) sniffing in psilocybin-treated female mice. Inset: psilocybin significantly increased DA release within the first 10 seconds of familiar sniffing (*p* = 0.0317), **J** but early DA levels (0-2 s) remained unchanged. **K** Average GRAB_DA2m_ traces recorded in male mice during the first sniff of a familiar or novel conspecific, 24 h after psilocybin or saline treatment. Inset: No significant effects of psilocybin on DA release were observed within the first 10 seconds of interaction. **L** At 24 h, psilocybin significantly reduced immediate (0–2 s) DA release during novel sniffing in males (*p* = 0.0386), with no effect on familiar sniffing. **M** Average GRAB_DA2m_ recordings from male mice during their first interaction with either a familiar or novel conspecific, 7 days post-administration of psilocybin or saline. Inset: No significant differences in total DA release were observed within 10 seconds post-interaction. **N** At 7 days, psilocybin significantly reduced immediate novelty-evoked DA release in males (*p* = 0.0326); saline-treated males showed greater DA release to novel versus familiar sniffing (*p* = 0.0353). Psilocybin (PSI), saline (SAL), dopamine (DA). Female and male subjects are represented in green and blue, respectively. Saline-treated animals are represented by dashed lines and open symbols, while psilocybin-treated animals are represented by solid lines and filled symbols. Data are presented as mean ± SEM. Statistical analyses were performed using unpaired t-test, one-way ANOVA or two-way ANOVA with Fisher’s LSD post hoc tests. *p < 0.05, **p < 0.01. Schematics created in BioRender.

Based on the dopamine (DA) system’s critical role in social motivation and exploratory behaviour, we examined DA dynamics in the NAc during barrier climbing (Fig. 5A) using fiber photometry to record GRAB_DA_-mediated fluorescence as a proxy for DA release (Fig. 5B). Since repeated interactions can attenuate DA release sensitivity, we focused our analysis on DA release during the first social interaction. Striatal DA was monitored in male and female mice at 24 h and 7 d following psilocybin or saline administration during social exploration involving an empty cage, a familiar or a novel mouse. When comparing DA release within the first 10 s of interaction, male mice exhibited significantly lower DA release than females when interacting with a novel mouse (*p* = 0.0082; Fig. 5C); however, no significant sex difference was observed during initial contact (first 2 s of sniffing; Fig. 5D).

To evaluate the effects of psilocybin, DA dynamics showed sex- and time-dependent effects. At 24 h post-administration, psilocybin did not affect DA release in female mice (Fig. 5E), neither the mean or peak DA release during the first 2 s after sniffing familiar or novel mice (Fig. 5F). In male mice, psilocybin did not affect DA release within the first 10 s of interaction with a familiar or novel mouse (Fig. 5G); however, it significantly reduced peak DA release during initial contact (0-2 s) with a novel mouse (*p* = 0.0386), without significantly altering overall and immediate average DA release (Fig. 5H). At 7 d post-administration, DA release in male and female mice was comparable within the first 10 s of interaction (Fig. 5I) and during initial contact (Fig. 5J). Psilocybin-treated females exhibited prolonged DA release elevation during the first interaction with the familiar mouse, but not the novel one (*p* = 0.0317, Fig. 5K) without a corresponding change in immediate (0–2 s) mean or peak DA release (Fig. 5L). In male mice, psilocybin did not affect total DA release at 7 d (Fig. 5M) but again significantly reduced immediate peak DA release during the first sniff of a novel mouse (*p* = 0.0326). Consistent with the view that novel conspecific interactions are more rewarding that familiar ones, saline-treated males showed significantly greater immediate peak DA release when sniffing a novel mouse compared to a familiar one (*p* = 0.0353; Fig. 5N), an effect absent in psilocybin-treated males.

## Discussion

This study demonstrates that psilocybin produces sex- and time-specific effects on social behaviour, mediated by distinct serotonergic and dopaminergic mechanisms. Rather than inducing uniform ‘prosocial’ outcomes, psilocybin modulated behaviour in a context- and sex-dependent manner, even in unstressed, socially housed mice, addressing a key gap in preclinical psychedelic research, where sex differences remain underexplored. We examined the acute and delayed effects of a single psilocybin dose on multiple aspects of sociability in male and female mice and assessed the involvement of 5-HT1AR and 5-HT2AR. We also measured dopamine release in the NAc, a key region within social reward circuits. Given the established interplay between serotonin and dopamine in regulating cognition and emotion [59], our findings contribute important insights into the neurobiological substrates underlying psychedelic action.

### Acute prosocial behaviour and temperature regulation

Male and female mice exhibited comparable baseline huddling behaviour; however, within 60 min of psilocybin administration, female mice showed pronounced increases in huddling behaviour, initially hypothesised to reflect thermoregulatory social bonding [60]. The absence of this effect in male mice may be related to previous reports of greater female sensitivity to the acute effects of psilocybin [61]. Importantly, reduced body temperature following psilocybin administration has previously been observed at higher doses of psilocybin (3 and 10 mg/kg) with no sex differences reported [62], arguing against a simple pharmacodynamic dose-equivalence explanation for our female-specific finding. Given that estrogen dynamically regulates both 5-HT2AR expression and serotonin availability across the estrous cycle [48], the 1.5 mg/kg dose may engage these sex-specific neurochemical states in a manner that produces female-specific behavioural responses that are qualitatively distinct rather than dose-shifted. The discrepancy between our temperature findings and the previous study [62], which reported no hypothermia at lower doses (0.03-1 mg/kg), likely arises from methodological differences: we continuously monitored temperature at 5-min intervals for 1 h post-administration using an automated system, allowing detection of transient fluctuations, whereas the previous study assessed temperature only once at 30 min, potentially overlooking dynamic thermoregulatory responses.

Crucially, the psilocybin-induced increase in huddling in females was abolished by both 5-HT1AR and 5-HT2AR antagonists, but this was not recapitulated in temperature measurements, indicating a more complex interplay between serotonergic signalling, huddling and temperature regulation. 5-HT1AR antagonism also reduced huddling *and* body temperature in saline-treated females, which again speaks to a dissociation between thermoregulation and social bonding even under control conditions. While effects of antagonists in saline-treated animals complicates interpretation of their role in psilocybin-mediated behaviours, the selective reduction in psilocybin-induced huddling by 5-HT2AR antagonism and distinct interaction patterns in males demonstrate receptor-specific modulation that extends beyond baseline effects. One alternative mechanism for the persistence of psilocybin-induced hypothermia even when 5-HT1AR or 5-HT2AR were antagonised is the 5-HT7R, which is implicated in thermoregulation and is widely expressed in the preoptic area (POA) of the hypothalamus, raphe nuclei and parabrachial nucleus, all of which are central to autonomic temperature control [63, 64]. Notably, 5-HT7R agonism induces hypothermia, and increased serotonergic tone following psilocybin administration may recruit this receptor [65–67]. Additionally, GABAergic and glutamatergic signalling within the POA may interact with serotonergic inputs to shape psilocybin’s hypothermic effects [68, 69].

Male mice did not exhibit psilocybin-induced huddling or changes in body temperature; however, at this acute time point, both 5-HT1AR and 5-HT2AR antagonists reduced aggression in saline-treated males, which was not observed with psilocybin co-administration. This suggests psilocybin engages broader serotonergic circuits that override receptor-specific modulation of aggression in males, contrasting with previous studies in which 5-HT1AR agonists suppressed aggression and antagonism reversed these effects [70]. These differing outcomes may reflect compensatory signalling or altered serotonergic tone in pair-housed animals compared to the socially isolated animals in earlier work. Sex differences in serotonergic architecture alongside recent evidence that MDMA-induced cortical plasticity in mice engages 5-HT2AR in a sex-dependent manner [71] may further explain sex-divergent behavioural responses to psilocybin observed in the present study. Women exhibit higher 5-HT1AR binding potential in multiple brain regions, though binding fluctuates with the menstrual cycle [72, 73] and this receptor is a central target of SSRIs in treating social anxiety and depression [74–76]. In contrast, men show higher cortical 5-HT2AR expression [72]. Similar patterns are observed in rodents, with female rats exhibiting higher 5-HT1AR signalling and males showing greater cortical 5-HT2AR expression [77], potentially explaining the differential involvement of these receptors in social and stereotypic behaviours in our study.

#### Social preference and exploration

Male mice showed a higher preference for social novelty than females during the social novelty trial, consistent with previous reports in the same paradigm [78]. Notably and consistent with previous reports [34, 36], psilocybin did not affect social preference in either sex at 24 h or 7 d post-administration. However, at 4 h, female mice showed increased grooming and social exploration, behaviours associated with both heightened anxiety and sociability in C57BL/6 mice [79]. These prosocial effects were abolished by both 5-HT1AR and 5-HT2AR antagonism, confirming involvement of these receptor subtypes. At 24 h, psilocybin reduced grooming and rearing in male mice, behaviours that may reflect anxiolytic effects or reduced behavioural responsiveness. While we did not assess whether changes in rearing or grooming were mediated by specific serotonin receptor subtypes in the present study, prior work has demonstrated that rearing, more frequent in males [80], is reduced by psilocin through 5-HT2AR mechanisms [81], while grooming is suppressed by SSRIs, 5-HT1AR agonists [82] and high doses of psilocybin or LSD [83–85].

Although earlier findings reported increased sociability in stressed female mice 24 h after psilocybin treatment [86], we did not observe similar prosocial effects at this time point in our unstressed mice. However, robust effects at other time points suggest that stress or social deficits are not necessary preconditions for psilocybin’s behavioural efficacy. While prior work showed that psilocybin can reverse social impairments in autism models without affecting controls [34], our data indicate that psilocybin can modulate social behaviour in healthy animals in a time- and context-dependent manner.

Our data support previous evidence that 5-HT1AR plays a key role in mediating the social effects of serotonergic compounds. WAY-100635 blocked psilocybin-induced prosocial behaviours, consistent with reports showing this antagonist prevented MDMA-induced increases in social interaction in male rats [87]. These findings suggest psilocybin initiates dynamic processes that unfold over time and differ between sexes. The persistence of effects beyond the acute pharmacological window may reflect underlying mechanisms such as neuroplastic changes, altered social learning or sustained serotonergic adaptations. The female-specific shift toward familiar social targets at later time points may indicate memory consolidation processes. Time-dependent effects could also be influenced by interactions between serotonergic and estrogen signalling [48]. Although we did not control for the estrous cycle in all behavioural tests, no significant correlation was observed between estrous stage and sociability in female mice (see Supplementary Methods and Supplementary Data 8). However, studies in female rats show that those in the estrous and proestrous phases (when estradiol and progesterone are highest) were less affected by psilocin and LSD compared to male rats [88, 89].

### Dopamine dynamics and social reward salience

NAc dopamine dynamics revealed a possible mechanistic substrate for sex-specific behavioural responses, considering that dopamine release in response to a novel social interaction was blunted in males compared to females. In females, psilocybin did not alter acute dopamine responses but induced a prolonged elevation in dopamine release during familiar, but not novel, interactions at 7 days post-administration. This may reflect enhanced social memory or encoding of familiar cues, consistent with findings that familiarity plays a larger role in female prosocial behaviour [90]. The psilocybin-induced increase in NAc dopamine during familiar interactions may reflect a recalibration of social reward circuits, wherein familiar social experiences become re-evaluated as uniquely rewarding within a new affective or cognitive context. The delayed response aligns with neuroplastic adaptations, such as increased spine density in the female mouse cortex post-psilocybin [91] and improved cognitive flexibility [92, 93]. These interactions underscore that NAc dopamine signals multiple dimensions, including salience, motivation, valence and prediction error. Psilocybin may recalibrate these behavioural computations in sex-specific ways.

In males, psilocybin reduced peak dopamine release during novel social encounters at both 24 h and 7 d, aligning with behavioural shifts toward familiar preference and reduced novelty salience. Saline-treated controls displayed the expected novelty-biased dopamine response [94], which was absent in psilocybin-treated males, suggesting psilocybin dampens novelty-driven dopaminergic activity, potentially reshaping the social reward landscape. Whether this reflects reduced novelty ‘appeal’ or increased reward value of familiar conspecifics remains uncertain. However, since the photometry-paired task did not require choosing between familiar and novel conspecifics, the latter explanation may be more plausible. Consistent with our observations, a recent study reported sex-specific responses, with psilocybin eliciting stronger behavioural effects and calcium transients in the paraventricular nucleus of female mice compared to males [95].

### Limitations and future directions

Some limitations warrant consideration: First, we only tested a single psilocybin dose, limiting conclusions about dose-response relationships. Second, our study focused on 5-HT1AR and 5-HT2AR, while other receptors like the 5-HT7R, 5-HT1BR and 5-HT2CR that regulate thermoregulation and social behaviour should be explored [64, 96]. We observed that 5-HT1AR antagonism reduced locomotor activity in female mice at 4 h, potentially reflecting sex-specific sensitivity to the antagonist dose, which complicates interpretation of the associated sociability findings. While this confound should be acknowledged, effects across other measures still offer novel insights into receptor-specific contributions, warranting further investigation. Additionally, although we discuss serotonergic-dopaminergic interactions, we did not assess the oxytocin system, which plays a central role in social recognition and bonding [97]. Finally, while fiber photometry is a powerful tool for monitoring dopamine dynamics, it captures only population-level changes and does not quantify absolute concentrations or resolve fine-scale spatial activity. These recordings do not clarify whether psilocybin’s effects arise from direct modulation of dopamine neuron activity in the midbrain or from local changes in the NAc, potentially mediated by 5-HT2AR signalling [98, 99]. They also do not distinguish whether altered dopamine dynamics are a consequence or a cause of sex differences in behaviour. Establishing causality will require approaches that temporally dissociate these variables, such as optogenetic control of ventral tegmental area dopamine neuron activity, pharmacological dopamine or serotonin receptor blockade or comparison to ovariectomized mice to isolate estrogen’s role in dopamine-behaviour coupling following psilocybin treatment.

Despite these limitations, this study is the first, to our knowledge, to assess the acute and delayed effects of psilocybin on multiple dimensions of social behaviour and dopamine release in male and female mice. The single-dose paradigm offers clearer interpretation, avoiding confounds from repeated administration and reflecting many current clinical contexts. The focus on 5-HT1AR and 5-HT2AR, two of the most well-characterised serotonin receptors, strengthens interpretability. We demonstrate that psilocybin modulates social behaviour and core body temperature through sexually dimorphic and temporally dynamic mechanisms, mediated by 5-HT1AR and 5-HT2AR signalling and NAc dopaminergic pathways. While 5-HT2AR has historically dominated psychedelic research, our results highlight the need to broaden this focus. These findings challenge assumptions of universal prosocial effects and underscore the critical importance of incorporating SABV in preclinical models and therapeutic development. Future studies should investigate broader receptor systems, hormonal influences and circuit-level mechanisms to further elucidate the complex, sex-specific neurobiology underlying psychedelic modulation of social cognition and behaviour.

## Supplementary information

Supplementary Materials

## Data Availability

All data available are presented in the main manuscript and additional supporting files. Raw data are available upon reasonable request to the corresponding author.
