# Supplementary material for: Psilocybin modulates social behaviour in male and female mice in a time-dependent manner
Source: Neuropsychopharmacology. 2026 May 25;51(9):1588–98. doi: 10.1038/s41386-026-02450-x (PMC13388970; doi:10.1038/s41386-026-02450-x)
Supplement: Supplementary file 1 — Supplementary Materials [file 41386_2026_2450_MOESM1_ESM.docx]

**Supplementary Methods (Detailed Protocols)**

**Animals and Housing - Extended Details**

All animals were obtained from the Monash Animal Research Platform (MARP; Clayton, VIC, Australia). Mice were pair-housed in a climate-controlled room (temperature 22-24°C and humidity 30-50%) on a reverse light cycle (lights on at 2000h, off at 0800h) for 7 days before experiments commenced for acclimation. Mice had ad libitum access to food and water, except during behavioural testing windows. Four sex- and age-matched C57Bl/6 mice were used per cohort as novel mice in the 3-chamber and barrier climbing tests and were housed in a separate room under the same conditions as test mice. All experimental procedures adhered to the Australian Code for the Care and Use of Animals for Scientific Purposes.

**Drug Preparation and Administration - Extended Details**

Psilocybin (USONA Institute Investigational Drug Supply Program; Lot# AMS0167) was dissolved in 0.9% NaCl saline. MDL100907 (volinanserin; Sigma-Aldrich, CAS 139290-65-6) and WAY100635 maleate (Tocris Biosciences, CAS 1092679-51-0) were also dissolved in saline. All drugs were delivered intraperitoneally with a 26-gauge needle at an injection volume of 10 ml/kg.

**Acute Core Body Temperature and Activity Monitoring - Extended Details**

The UID Temperature Monitoring System (Unified Information Devices, Kenosha, WI, USA) was used to measure digital biomarkers continuously 24/7 from conscious, unrestrained mice in their home-cage environment. This approach reduced stress and ensured reliable data collection through a temperature-sensitive microchip in an undisturbed setting. Upon arrival, pair-housed mice were subcutaneously implanted with a 2.1 mm x 13 mm microchip which uses RFID technology for animal identification and body temperature monitoring (model UCT-2112), implanted via a specialized injection device.

**3-Chamber Test - Extended Details**

Mice were placed in the central chamber of an apparatus containing three chambers, each with a wire cage at either end, for a 10-minute habituation period. A 1 min inter-trial interval (ITI) followed, during which mice were confined to the middle chamber to minimize experimenter interference. Subsequently, an unfamiliar, age- and sex-matched mouse (Novel 1) was placed in a wire cage in one side chamber, while a novel object in a wire cage occupied the opposite chamber. The test mouse was then allowed to explore for 10 min (social preference trial). After another 1 min ITI, a second unfamiliar age- and sex-matched mouse (Novel 2) replaced the novel object, with Novel 1 remaining as the familiar mouse. The test mouse was observed for another 10 min (social novelty trial). Locomotor activity, interaction frequency, time spent in each chamber, and social preference were measured using video tracking and EthoVision XT software. The Sociability Index measures relative time spent interacting with either a novel conspecific, an empty cage, or a familiar mouse. Stereotypic behaviours assessed included grooming, rearing, cage climbing, and freezing.

**Barrier Climbing Test - Extended Details**

Behaviour was recorded for a total of 20 min, including habituation to the barrier (10 min), exploration of an unfamiliar age- and sex-matched mouse (5 min), and exploration of a cagemate (5 min). The novel mouse and cagemate were placed in a wire cage on the opposite side of the barrier to the test mouse. A barrier climb was defined as the moment when all four paws of the mouse touched the floor on opposite side of the 60 mm transparent barrier. Each instance of the mouse crossing the barrier and placing all four paws on the opposite side was counted as a barrier climb.

**Estrous Cycle Assessment**

Vaginal smears were collected daily at 1000h over a period of 7 d prior to testing and on test days following procedures. A volume of 10 µL of saline was flushed into the vagina, transferred onto a glass slide, and examined as stained preparations using hematoxylin and eosin (H&E). The stage of the estrous cycle was determined and classified as proestrus, estrus, or metestrus/diestrus, based on observed ratios of cornified epithelial, nucleated epithelial, and polymorphonuclear leukocytes [1].

**Surgical and Viral Injection Procedures - Extended Details**

Mice were anesthetized with isoflurane (2-3%; Pharmachem, QLD, Australia) and administered meloxicam (5 mg/kg, Boehringer Ingelheim, Germany) subcutaneously prior to surgery. Each animal was positioned in a stereotaxic frame (Stoelting, IL, USA) on a heating pad maintained at 37.2°C. Mice were unilaterally injected in the right hemisphere with hsyn-GRABDA2m for dopamine recordings. A volume of 300 nL was injected at a rate of 30 nL/min using a 2 μL Hamilton syringe (Model #7002), and the needle was left in place for 5 min post-infusion to allow for diffusion. Optical fibers (RWD, 0.39 NA; length: 4.7 mm; core: 200 nm) were implanted 0.1 mm above the injection site (final DV: -4.1 mm) and secured using bonding agent (GBond, Japan) and light-cured dental cement (G-aenial Universal Flow, GC Dental).

**Fiber Photometry - Extended Details**

Experiments began at least five weeks post-surgery to allow sufficient time for recovery and viral expression. Recordings were conducted using the RWD R821 fiber photometry system, with 470 nm and 410 nm lasers used for the signal and isosbestic control channels, respectively. Data acquisition was performed using RWD software during the barrier climbing task, and behavioural video timestamps were manually aligned to the photometry recordings.

**Data Analysis - Extended Details**

A significance threshold of p < 0.05 was applied, while p < 0.10 was considered indicative of a trend, though not statistically significant. Depending on the data type, number of groups, and comparisons of interest, appropriate statistical methods were used, including two-tailed unpaired t-tests, one-way and two-way analyses of variance (ANOVA) with Sidak's post hoc multiple comparisons and mixed-effects models.

**References**

1. Nelson, J.F., et al., *A longitudinal study of estrous cyclicity in aging C57BL/6J mice: I. Cycle frequency, length and vaginal cytology.* Biol Reprod, 1982. **27**(2): p. 327-39.


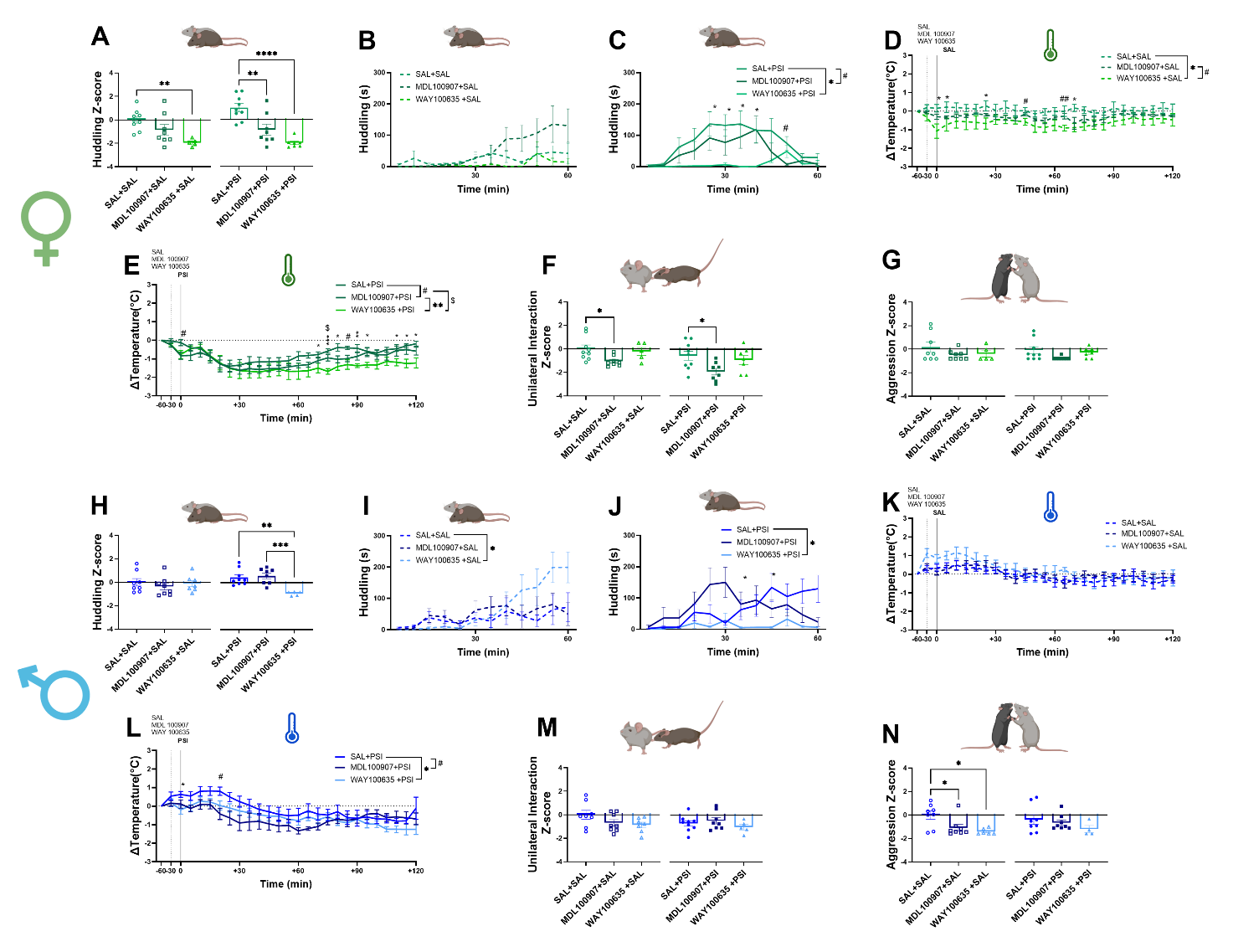


**Supplementary Data 1. 5-HT2AR and 5-HT1AR antagonists differentially affect huddling and unilateral interaction in male and female mice. (A)** Among saline-treated mice, 5-HT1AR antagonism reduced huddling behaviour (*p* = 0.0062), and in psilocybin-treated mice, both 5-HT2AR (*p* = 0.0029) and 5-HT1AR (*p* < 0.0001) antagonists reduced huddling behaviour. **(B)** 5-HT2AR or 5-HT1AR antagonism did not significantly affect huddling behaviour over the 60-minute observation period in saline-treated mice; **(C)** however, in psilocybin-treated mice, 5-HT1AR antagonism reduced huddling behaviour at 25 min (*p* = 0.0228), 30 min (*p* = 0.0158), 35 min (*p* = 0.0275), and 40 min (*p* = 0.0428), while 5-HT2AR antagonism reduced huddling behaviour only at 50 min (*p* = 0.0419) compared to the psilocybin-treated group. **(D)** In saline-treated mice, 5-HT1AR antagonism lowered core body temperature at 0 min (*p* = 0.0206), 5 min (*p* = 0.0398), 25 min (*p* = 0.0257), 65 min (*p* = 0.027), and 70 min (*p* = 0.0112) compared to the saline-treated group, and produced a stronger temperature reduction than 5-HT2AR antagonism at 45 min (*p* = 0.0258) and 65 min (*p* = 0.0099). **(E)** In psilocybin-treated mice, 5-HT1AR antagonism reduced core body temperature at 75 min (*p* = 0.021), while 5-HT2AR antagonism increased temperature at 0 min (*p* = 0.0398) and 85 min (*p* = 0.0429) compared to the psilocybin-treated group. Additionally, 5-HT1AR antagonism produced significantly lower body temperature than 5-HT2AR antagonism between 75 and 120 minutes (time x treatment interaction: *F* (2,22) = 2.868, *p* = 0.0152). **(F)** In saline-treated female mice, 5-HT2AR antagonism significantly reduced the unilateral interaction (*p* = 0.0339) and a similar reduction was observed in psilocybin-treated females (*p* = 0.0328). **(G)** Neither psilocybin nor 5-HT receptor antagonism significantly affected aggressive behaviour in female mice. **(H)** In saline-treated male mice, 5-HT2AR or 5-HT1AR antagonism did not significantly affect the huddling behaviour. However, in psilocybin-treated mice, 5-HT1AR antagonism significantly reduced huddling behaviour compared to psilocybin only group (*p* = 0.0018) and compared to the 5-HT2AR antagonism (*p* = 0.0009). **(I)** No significant differences in huddling behaviour were observed over 60 minutes among saline-treated groups. **(J)** In psilocybin-treated mice, 5-HT1AR antagonism reduced huddling behaviour at 45 min (*p* = 0.0496) and 55 min (*p* = 0.0489). **(K)** Core body temperature did not differ significantly among saline-treated groups over 60 minutes. **(L)** In psilocybin-treated mice, 5-HT1AR reduced core body temperature at 0 min (*p* = 0.0289) and 5-HT2AR antagonism also lowered core body temperature at 20 min (*p* = 0.0165). **(M)** No significant effects of psilocybin, 5-HT1AR antagonism, or 5-HT2AR antagonism were observed on unilateral interaction in male mice. **(N)** In saline-treated male mice, both 5-HT1AR (*p* = 0.0101) and 5-HT2AR (*p* = 0.0366) antagonism significantly reduced aggressive behaviour. Psilocybin (PSI), saline (SAL), 5-HT2AR antagonist (MDL100907), 5-HT1AR antagonist (WAY100635). Female and male subjects are represented in green and blue, respectively. Saline-treated animals are represented by dashed lines and open symbols, while psilocybin-treated animals are represented by solid lines and filled symbols. Z-score = normalised measure relative to saline-treated animals. Data are presented as mean ± SEM and were analysed using one-way ANOVA with Šidák post hoc tests. **p* < 0.05, ^#^*p*< 0.05, ^$^*p*< 0.05, ***p*< 0.01, ^##^*p*< 0.01, ****p*< 0.001, *****p*< 0.0001.


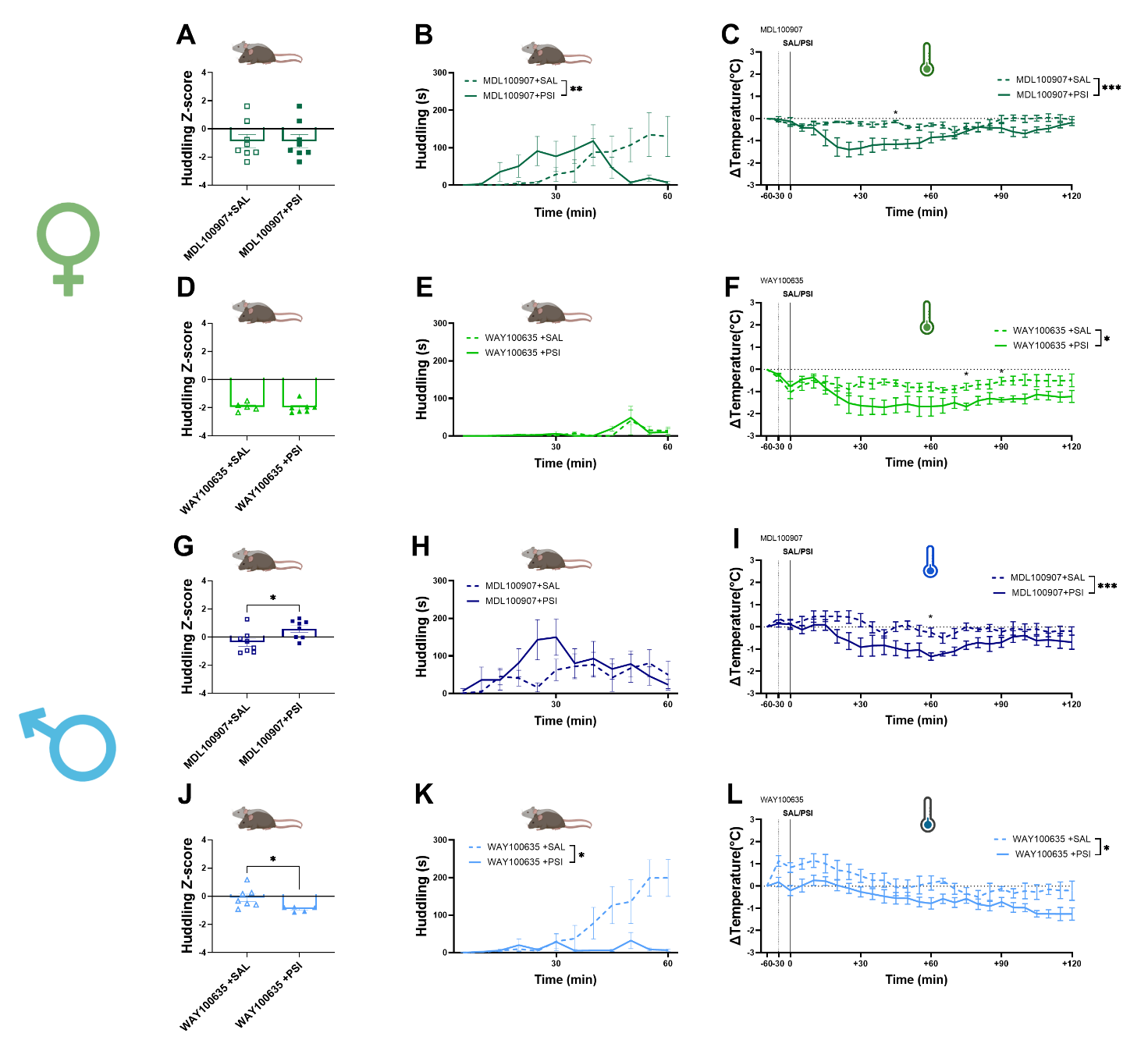


**Supplementary Data 2. Psilocybin reduces core body temperature irrespective of 5-HT2AR or 5-HT1AR antagonism but differentially affects huddling behaviour in male mice. (A)** Total huddling behaviour was not affected by psilocybin in mice pre-treated with the 5-HT2AR antagonist, **(B)** nor were any time-dependent differences in huddling observed throughout the 60-minute trial. **(C)** However, psilocybin significantly reduced core body temperature in this group at 45 min post-administration (*p* = 0.0297). **(D)** Similarly, total huddling behaviour was unaffected by psilocybin in mice pre-treated with the 5-HT1AR antagonist. **(E)** No time-dependent changes in huddling were observed across the 60-minute observation period. **(F)** In contrast, psilocybin significantly reduced core body temperature in these mice at 75 min (*p* = 0.0446) and 90 min (*p* = 0.0478) post-administration. **(G)** In male mice pre-treated with the 5-HT2AR antagonist, psilocybin increased total huddling behaviour (*p* = 0.0194). **(H)** However, no significant differences were observed in huddling behaviour across the 60-minute trial. **(I)** Psilocybin significantly reduced core body temperature in this group at 60 minutes post-administration (*p* = 0.0365). **(J)** In mice pre-treated with the 5-HT1AR antagonist, psilocybin significantly reduced total huddling behaviour compared to controls (*p* = 0.0327). **(K)** No significant changes in huddling behaviour were observed across the 60-minute period. **(L)** Psilocybin did not significantly alter core body temperature in mice pre-treated with the 5-HT1AR antagonist. Psilocybin (PSI), saline (SAL), 5-HT2AR antagonist (MDL100907), 5-HT1AR antagonist (WAY100635). Female and male subjects are represented in green and blue, respectively. Saline-treated animals are represented by dashed lines and open symbols, while psilocybin-treated animals are represented by solid lines and filled symbols. Z-score = normalised measure relative to saline-treated animals. Data are presented as mean ± SEM. Statistical analyses were performed using unpaired t-test, mixed-effects models or two-way ANOVA with Šidák post hoc tests. **p*< 0.05, ***p*< 0.01, ****p*< 0.001.


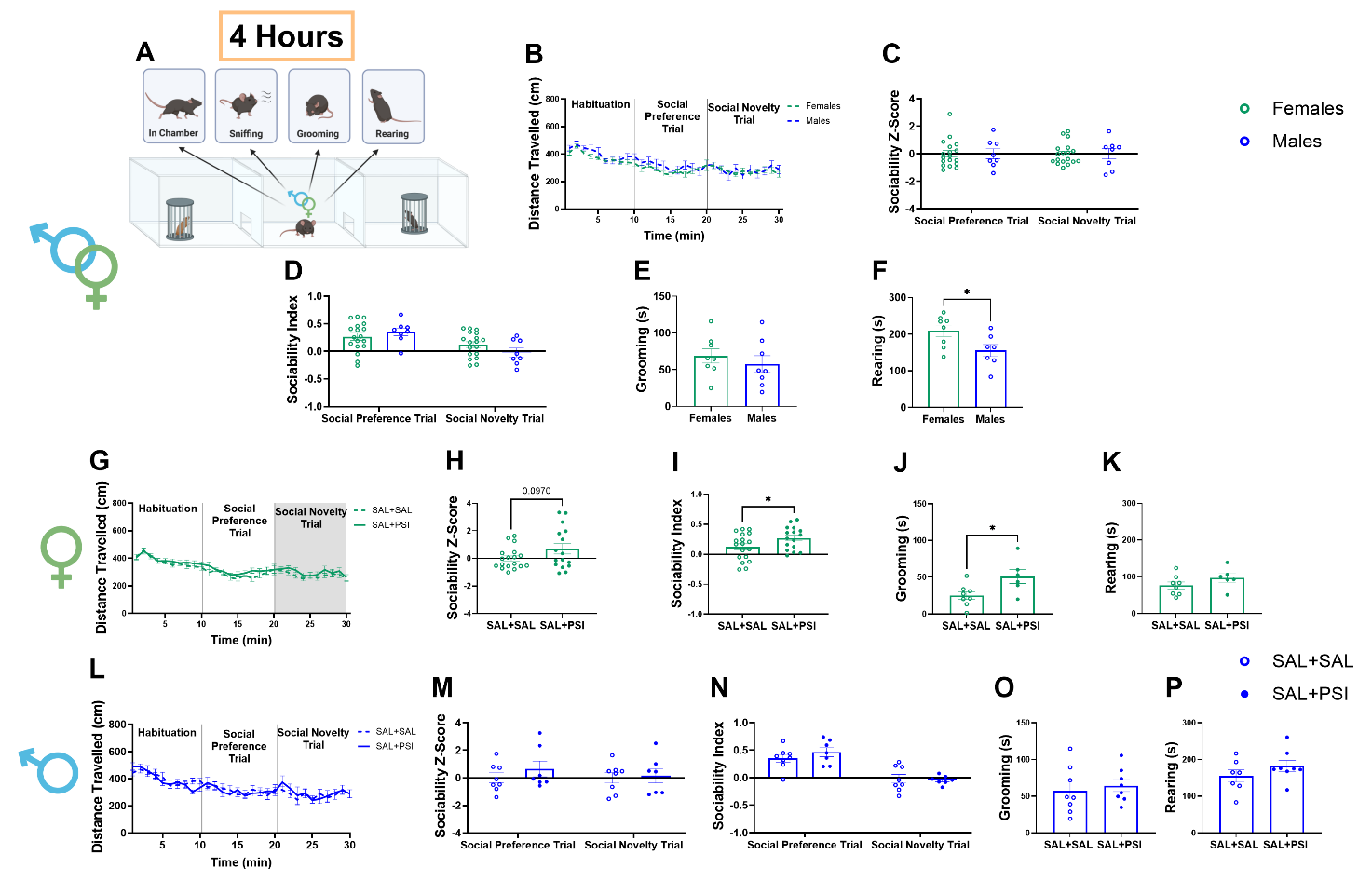


Supplementary Data 3. At 4 hours post-administration, psilocybin selectively enhances sociability and grooming behaviours in female but not male mice. (A) Schematic of the social novelty trial and the behaviours scored. (B) Baseline locomotor activity did not differ between male and female mice, nor did their (C, D) sociability measures during the social preference and novelty trials. (E) Grooming behaviour did not differ significantly between sexes; however, (F) females exhibited significantly higher rearing behaviour throughout the experiment (*p* = 0.0333). (G) Psilocybin did not alter locomotor activity of female mice across the duration of the experiment. (H) The total sociability Z-score of psilocybin-treated mice approached significance compared to controls (*p* = 0.097). (I) Psilocybin-treated mice displayed significantly higher sociability during the social novelty trial compared to controls (*p* = 0.0494). (J) Psilocybin increased grooming behaviour (*p* = 0.0443), while (K) rearing behaviour was unaffected. (L) In male mice, psilocybin had no effect on locomotor activity, (M) sociability Z-score, (N) direct sociability, (O) grooming or (P) rearing. Psilocybin (PSI), saline (SAL). Female and male subjects are represented in green and blue, respectively. Saline-treated animals are represented by dashed lines and open symbols, while psilocybin-treated animals are represented by solid lines and filled symbols. Z-score = normalised measure relative to saline-treated animals; sociability index = relative direct interaction time between the social and non-social stimuli. Data are presented as mean ± SEM. Statistical analyses were performed using unpaired t-test or two-way ANOVA with Šidák post hoc tests. **p* < 0.05.


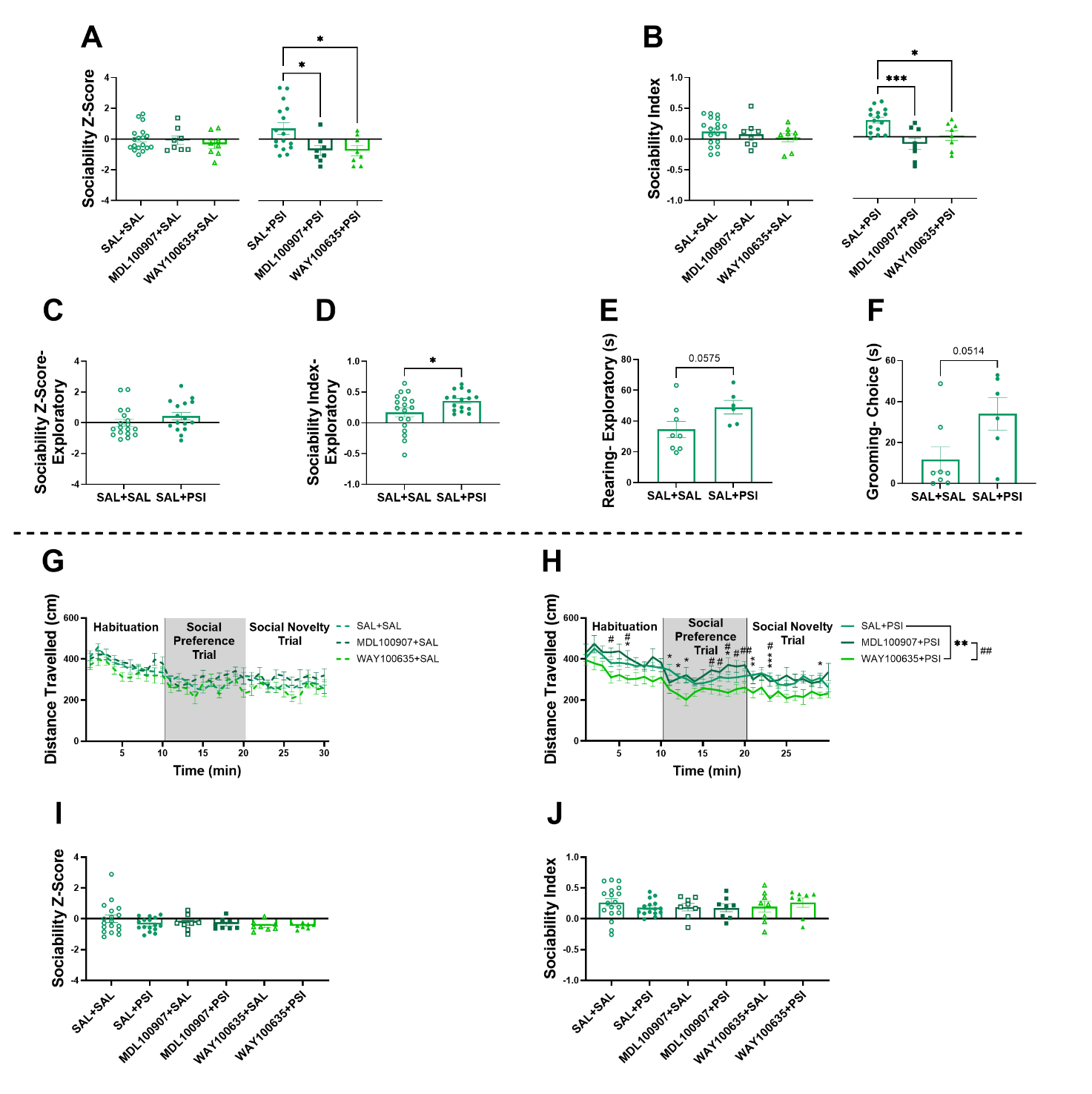


**Supplementary Data 4. Psilocybin alters social behaviour in female mice in a trial-dependent manner and 5-HT2AR and 5-HT1AR antagonism modulates locomotion at 4 hours post-administration. (A)** In saline-treated mice, 5-HT1AR and 5-HT2AR antagonism did not affect sociability. In contrast, in psilocybin-treated mice, both 5-HT1AR (*p* = 0.023) and 5-HT2AR (*p* = 0.0252) antagonism significantly reduced sociability Z-score. **(B)** Similarly, 5-HT1AR and 5-HT2AR antagonism did not affect sociability in saline-treated mice, but in psilocybin-treated mice, both 5-HT1AR (*p* = 0.0243) and 5-HT2AR (*p* = 0.0007) antagonism reduced sociability. **(C)** Sociability Z-score was not significantly altered by psilocybin treatment; **(D)** however, psilocybin-treated mice displayed increased sociability toward the novel mouse compared to familiar conspecifics (*p* = 0.0381). **(E)** These mice also showed a non-significant trend toward increased grooming behaviour during the choice phase (*p* = 0.0514), and **(F)** a non-significant trend toward increased rearing behaviour during the exploratory phase (*p* = 0.0575). **(G)** 5-HT1AR and 5-HT2AR antagonism alone did not significantly affect behaviour across the trial duration. **(H)** In psilocybin-treated mice, pre-treatment with 5-HT1AR antagonist significantly reduced locomotor activity compared to the psilocybin-only group at multiple time points: 6 min (*p* = 0.0227), 11 min (*p* = 0.0188), 12 min (*p* = 0.0123), 13 min (*p* = 0.0256), 18 min (*p* = 0.0236), 21 min (*p* = 0.039), 23 min (*p* = 0.0006), and 29 min (*p* = 0.0165). Compared to the 5-HT2AR antagonist group, the 5-HT1AR antagonist also significantly reduced locomotion at: 4 min (*p* = 0.0107), 6 min (*p* = 0.0465), 16 min (*p* = 0.0297), 17 min (*p* = 0.0278), 18 min (*p* = 0.0151), 19 min (*p* = 0.032), 20 min (*p* = 0.0068), and 23 min (*p* = 0.0329). **(I)** During the social preference trial, neither psilocybin nor 5-HT1AR or 5-HT2AR antagonism significantly affected the sociability Z-score, **(J)** nor did they affect direct sociability measures. Psilocybin (PSI), saline (SAL), 5-HT2AR antagonist (MDL100907), 5-HT1AR antagonist (WAY100635). Saline-treated animals are represented by dashed lines and open symbols, while psilocybin-treated animals are represented by solid lines and filled symbols. Z-score = normalised measure relative to saline-treated animals; sociability index = relative direct interaction time between the social and non-social stimuli. Data are presented as mean ± SEM. Statistical analyses were performed using unpaired t-test, one-way ANOVA or two-way ANOVA with Šidák post hoc tests. **p*< 0.05, ***p*< 0.01, ^##^*p*< 0.01, ****p*< 0.001.

**
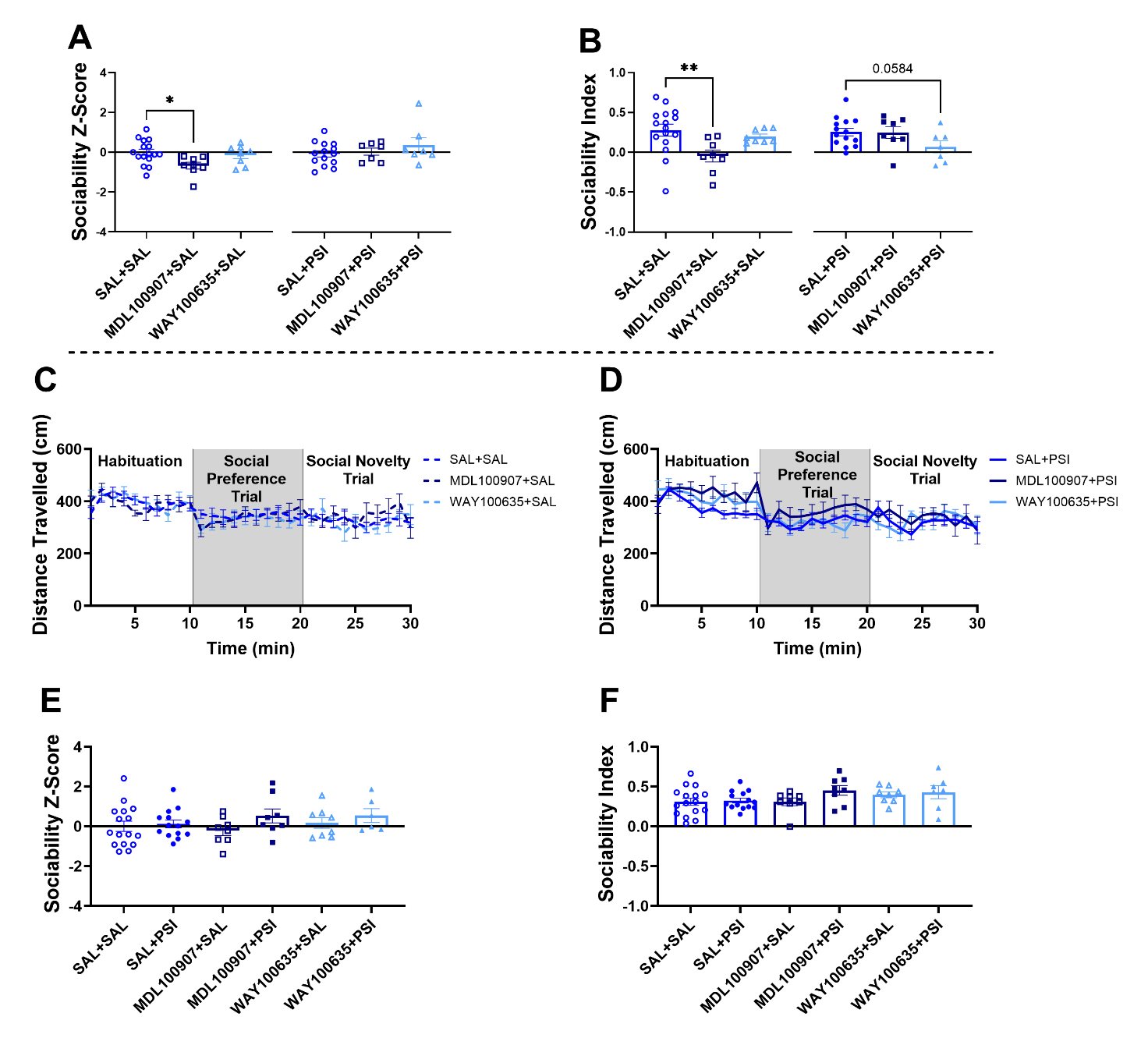
**

**Supplementary Data 5. In male mice 5-HT2AR and 5-HT1AR antagonism alter sociability and locomotion in a trial-dependent manner at 24 hours. (A)** In saline-treated mice, 5-HT2AR antagonism reduced sociability Z-score (*p* = 0.0168). Neither 5-HT1AR nor 5-HT2AR antagonism affected sociability in psilocybin-treated mice. **(B)** Similarly, 5-HT2AR antagonism reduced sociability in saline-treated mice (*p* = 0.0077), while psilocybin-treated mice pre-treated with the 5-HT1AR antagonist showed a non-significant trend toward reduced sociability (*p* = 0.0584). **(C)** 5-HTR antagonism did not alter locomotor activity in saline-treated male mice, **(D)** nor did it alter locomotor activity in psilocybin-treated males. **(E)** Neither psilocybin nor 5-HT1AR or 5-HT2AR antagonism affected the sociability Z-score of male mice during the social preference trial, (**F**) and no differences in overall sociability were observed during the same trial. Psilocybin (PSI), saline (SAL), 5-HT2AR antagonist (MDL100907), 5-HT1AR antagonist (WAY100635). Saline-treated animals are represented by dashed lines and open symbols, while psilocybin-treated animals are represented by solid lines and filled symbols. Z-score = normalised measure relative to saline-treated animals; sociability index = relative direct interaction time between the social and non-social stimuli. Data are presented as mean ± SEM. Statistical analyses were performed using one-way ANOVA or two-way ANOVA with Šidák post hoc tests. **p*< 0.05, ***p*< 0.01.


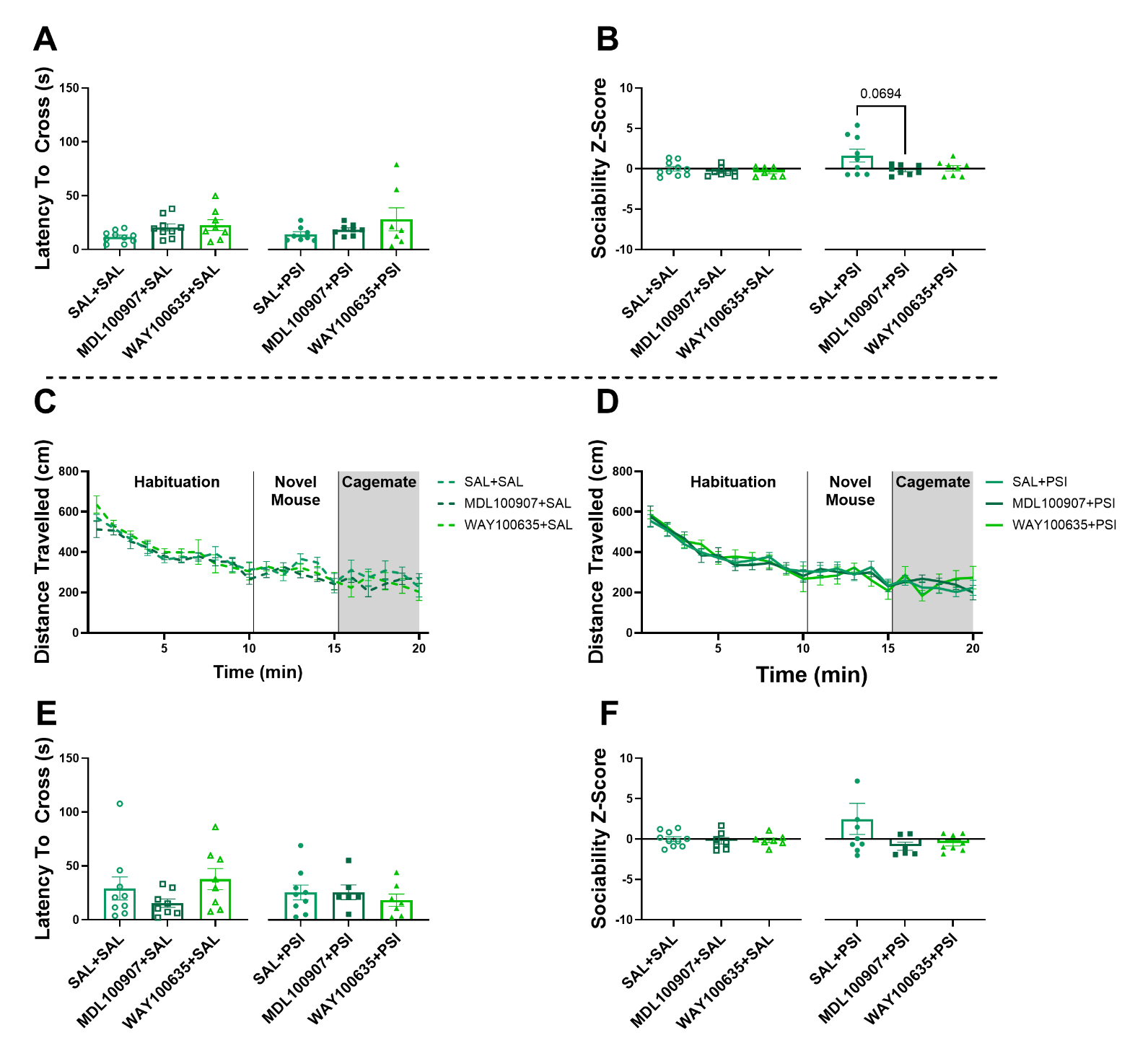


**Supplementary Data 6. Psilocybin, 5-HT2AR and 5-HT1AR antagonism do not affect locomotor activity or sociability in female mice during the barrier climbing test at 24 hours post-administration. (A)** 5-HT1AR and 5-HT2AR antagonism did not significantly alter barrier crossing latency. **(B)** Sociability Z-score when exploring the novel conspecific was similarly unaffected; however, in psilocybin-treated mice, mice pre-treated with the 5-HT2AR antagonist showed a non-significant trend toward reduced sociability (*p* = 0.0694). **(C)** 5-HT1AR and 5-HT2AR antagonism did not alter locomotor activity in saline-treated female mice, **(D)** nor did it alter locomotor activity in psilocybin-treated females. **(E)** Neither psilocybin nor 5-HT1AR or 5-HT2AR antagonism affected the latency to cross the barrier, **(F)** nor did they affect sociability in female mice. Psilocybin (PSI), saline (SAL), 5-HT2AR antagonist (MDL100907), 5-HT1AR antagonist (WAY100635). Saline-treated animals are represented by dashed lines and open symbols, while psilocybin-treated animals are represented by solid lines and filled symbols. Z-score = normalised measure relative to saline-treated animals. Data are presented as mean ± SEM. Statistical analyses were performed using one-way ANOVA or two-way ANOVA with Šidák post hoc tests.

**
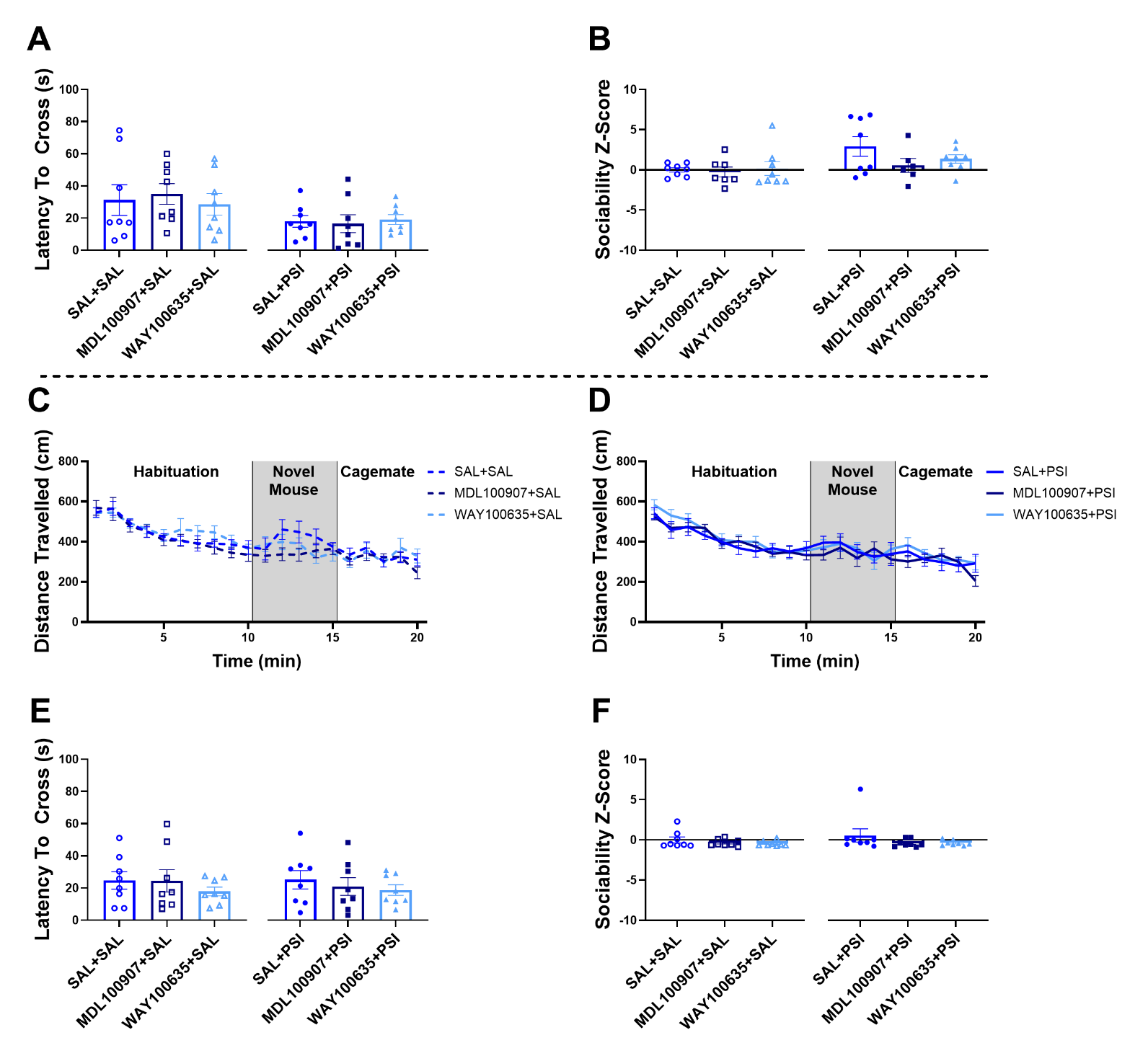
**

**Supplementary Data 7. Psilocybin, 5-HT2AR and 5-HT1AR antagonism do not affect locomotor activity or sociability in male mice during the barrier climbing test at 24 hours post-administration. (A)** 5-HT1AR and 5-HT2AR antagonism, alone or in combination with psilocybin, did not significantly affect latency to cross the barrier, **(B)** nor sociability when exploring cage mates. **(C)** 5-HT1AR and 5-HT2AR antagonism did not affect locomotor activity in saline-treated male mice, **(D)** nor did it affect locomotor activity in psilocybin-treated males. **(E)** No effects of psilocybin, 5-HT1AR antagonism, or 5-HT2AR antagonism were observed on latency to cross the barrier toward a novel mouse, **(F)** nor on sociability toward the novel mouse in male mice. Psilocybin (PSI), saline (SAL), 5-HT2AR antagonist (MDL100907), 5-HT1AR antagonist (WAY100635). Saline-treated animals are represented by dashed lines and open symbols, while psilocybin-treated animals are represented by solid lines and filled symbols. Z-score = normalised measure relative to saline-treated animals. Data are presented as mean ± SEM. Statistical analyses were performed using one-way ANOVA or two-way ANOVA with Šidák post hoc tests.


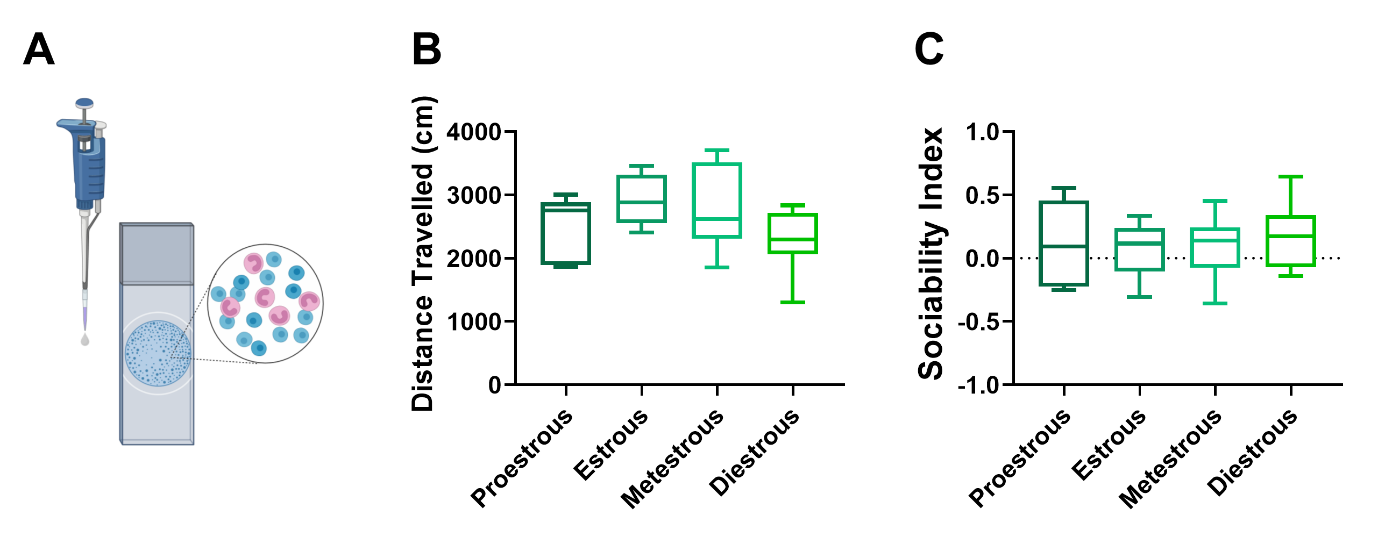


**Supplementary Data 8. No correlation was observed between the stage of the estrous cycle and either locomotor activity or sociability in female mice during the social novelty trial. (A)** Schematic representation of vaginal smear collection, with representative histological images of cell types observed under the microscope. **(B)** The locomotion and **(C)** sociability index did not differ significantly across estrous cycle stages. Data are presented as mean ± SEM. Statistical analysis was performed using one-way ANOVA.

## **Statistics Table Figure 1.**

| Figure | Statistical test | Group n | Main analysis result | Post-hoc multiple comparisons of interest |
| --- | --- | --- | --- | --- |
| 1.A | t-test | Females = 8  Males = 8 | N/A | N/A |
| 1.B | Two-way ANOVA | Females = 9  Males = 6 | N/A | N/A |
| 1.C | Mixed-effects analysis | Females = 9  Males = 8 | Time x sex interaction (F (5.541,81.62) = 2.833, p = 0.0173) | N/A |
| 1.D | t-test | SAL+SAL = 9  SAL+PSI = 9 | t (15.47) = 2.253, p= 0.0391 | N/A |
| 1.E | Two-way ANOVA | SAL+SAL = 9  SAL+PSI = 9 | Time (F (4.598, 72.32) = 3.136, p= 0.0151); Treatment (F (1, 16) = 7.723, p= 0.0134)  Time x treatment interaction (F (4.598, 72.32) = 3.105, p= 0.0159) | 25 min (= 0.0133), 30 min (p= 0.0155), 45 min (p=0.0311) |
| 1.F | Mixed-effects analysis | SAL+SAL = 9  SAL+PSI = 9 | Time (F (26, 394) = 7.185, p< 0.0001); Treatment (F (1, 16) = 16.1, p= 0.0001)  Time x treatment interaction (F (26, 394) = 5.626, p<0.0001) | 5 min (p= 0.0277), 15 min (p=0.0277), 20 min (p= 0.0004), 25 min (p< 0.0001), 30 min (p< 0.0001), 35 min (p< 0.0001), 40 min (p< 0.0001), 45 min (p< 0.0001), 50 min (p= 0.0001), 55 min (p= 0.0018), 60 min (p= 0.0111), 65 min (p= 0.0026), 70 min (p= 0073) |
| 1.G | t-test | SAL+SAL = 8  SAL+PSI = 9 | N/A | N/A |
| 1.H | Two-way ANOVA | SAL+SAL = 8  SAL+PSI = 9 | Time (F (11,143) = 3.923, p< 0.0001) | N/A |
| 1.I | Mixed-effects analysis | SAL+SAL = 8  SAL+PSI = 9 | Time (F (4.092,56.19) = 10.65, p< 0.0001) | N/A |

## **Statistics Table Figure 2.**

| Figure | Statistical test | Group n | Main analysis result | Post-hoc multiple comparisons of interest |
| --- | --- | --- | --- | --- |
| 2.B | Two-way ANOVA | Females = 7  Males = 12 | Sex x time interaction (F (9.42,197.8) = 2.217, p = 0.0206) | N/A |
| 2.C | Two-way ANOVA | Females = 7  Males = 12 | N/A | N/A |
| 2.D | Two-way ANOVA | Females = 7  Males = 12 | Sex (F (1,42) = 5.711, p=0.0214) | Social Novelty Trial (p = 0.0341) |
| 2.E | t-test | Females = 7  Males = 7 | t (8.308) = 1.891, p =0.094 | N/A |
| 2.F | t-test | Females = 7  Males = 7 | N/A | N/A |
| 2.G | Two-way ANOVA | SAL+SAL = 7  SAL+PSI = 8 | N/A | N/A |
| 2.H | Two-way ANOVA | SAL+SAL = 7  SAL+PSI = 8 | N/A | N/A |
| 2.I | Two-way ANOVA | SAL+SAL = 7  SAL+PSI = 8 | N/A | N/A |
| 2.J | t-test | SAL+SAL = 7  SAL+PSI = 8 | N/A | N/A |
| 2.K | t-test | SAL+SAL = 7  SAL+PSI = 8 | N/A | N/A |
| 2.L | Two-way ANOVA | SAL+SAL = 15, SAL+PSI = 16 | Time (F (10.09,282.5) = 8.968, p< 0.0001) | N/A |
| 2.M | Two-way ANOVA | SAL+SAL = 16, SAL+PSI = 14 | N/A | N/A |
| 2.N | Two-way ANOVA | SAL+SAL = 16, SAL+PSI = 14 | N/A | N/A |
| 2.O | t-test | SAL+SAL = 7, SAL+PSI = 8 | t (7.119) = 3.145, p= 0.0159 | N/A |
| 2.P | t-test | SAL+SAL = 7, SAL+PSI = 8 | t (10.8) = 3.744, p= 0.0033 | N/A |

## **Statistics Table Figure 3.**

| Figure | Statistical test | Group n | Main analysis result | Post-hoc multiple comparisons of interest |
| --- | --- | --- | --- | --- |
| 3.B | Two-way ANOVA | Females = 8, Males = 8 | Time (F (6.096,85.34) = 15.24, p< 0.0001)  Time x sex interaction (F (6.096,85.34) = 4.73, p = 0.0003) | 1 min (p = 0.0064)  5 min (p = 0.0256) |
| 3.C | t-test | Females = 8, Males = 8 | N/A | N/A |
| 3.D | t-test | Females = 8, Males = 8 | N/A | N/A |
| 3.E | t-test | Females = 8, Males = 8 | N/A | N/A |
| 3.F | t-test | Females = 8, Males = 8 | t (14) = 7.183, p< 0.0001 | N/A |
| 3.G | Two-way ANOVA | SAL+SAL = 8, SAL+PSI = 8 | Time (F (5.858,70.3) = 19.07, p< 0.0001 | N/A |
| 3.H | Two-way ANOVA | SAL+SAL = 8, SAL+PSI = 8 | Treatment (F (1,14) = 4.967, p = 0.0427  Treatment x trial interaction (F (1,14) = 7.406, p = 0.0165) | p = 0.0031 |
| 3.I | Two-way ANOVA | SAL+SAL = 8, SAL+PSI = 8 | N/A | N/A |
| 3.J | t-test | SAL+SAL = 8, SAL+PSI = 8 | N/A | N/A |
| 3.K | t-test | SAL+SAL = 8, SAL+PSI = 8 | N/A | N/A |
| 3.L | Two-way ANOVA | SAL+SAL = 8, SAL+PSI = 8 | Time (F (8.208,114.9) = 8.648, p< 0.0001 | N/A |
| 3.M | Two-way ANOVA | SAL+SAL = 8, SAL+PSI = 8 | N/A | N/A |
| 3.N | Two-way ANOVA | SAL+SAL = 8, SAL+PSI = 8 | N/A | N/A |
| 3.O | t-test | SAL+SAL = 8, SAL+PSI = 8 | t (14) = 2.464, p< 0.0273 | N/A |
| 3.P | t-test | SAL+SAL = 8, SAL+PSI = 8 | N/A | N/A |

## **Statistics Table Figure 4.**

| Figure | Statistical test | Group n | Main analysis result | Post-hoc multiple comparisons of interest |
| --- | --- | --- | --- | --- |
| 4.B | Mixed-effects analysis | Females = 10  Males = 8 | Sex (F (1,16) = 5.992, p = 0.0263) | N/A |
| 4.C | Two-way ANOVA | Females = 10  Males = 8 | Phase (F (1.511,23.42) = 11.08, p = 0.001 | Habituation (p = 0.0443) |
| 4.D | t-test | Females = 10  Males = 8 | N/A | N/A |
| 4.E | t-test | Females = 10  Males = 8 | N/A | N/A |
| 4.F | Two-way ANOVA | SAL+SAL = 10, SAL+PSI = 9 | Time (F (6.184,102.8) = 20.08, p< 0.0001) | N/A |
| 4.G | Two-way ANOVA | SAL+SAL = 10, SAL+PSI = 8 | N/A | N/A |
| 4.H | t-test | SAL+SAL = 10, SAL+PSI = 9 | t (17) = 2.045, p= 0.0566 | N/A |
| 4.I | Two-way ANOVA | SAL+SAL = 8, SAL+PSI = 8 | Time (F (6.424,89.6) = 10.81, p< 0.0001) | N/A |
| 4.J | Two-way ANOVA | SAL+SAL = 8, SAL+PSI = 8 | Phase (F (2,42) = 18.77, p< 0.0001) | Habituation (p= 0.0407) |
| 4.K | t-test | SAL+SAL = 8, SAL+PSI = 8 | t (14) = 2.335, p= 0.0349 | N/A |

## **Statistics Table Figure 5.**

| Figure | Statistical test | Group n | Main analysis result | Post-hoc multiple comparisons of interest |
| --- | --- | --- | --- | --- |
| 5.C | Mixed-effects analysis  Inset: Two-way ANOVA | Females = 5  Males = 5 | Familiar Mouse: N/A  Novel Mouse: N/A  Inset: F (1,7) = 12.64, p = 0.0093 | Inset: Novel Mouse: p= 0.0082 |
| 5.D | Two-way ANOVA | Females = 5  Males = 4 | Peak Z-Score: Sex (F (1,7) = 6.264, p= 0.0408) | N/A |
| 5.E | Mixed-effects analysis  Inset: Two-way ANOVA | SAL = 5, PSI= 5 | N/A | N/A |
| 5.F | Two-way ANOVA | SAL = 5, PSI= 5 | N/A | N/A |
| 5.G | Two-way ANOVA | SAL = 4, PSI= 3 | N/A | N/A |
| 5.H | Two-way ANOVA | SAL = 4, PSI= 3 | Peak Z-Score: Treatment (F (1,10) = 4.994, p= 0.0494) | Novel Mouse: SAL vs PSI: p= 0.086 |
| 5.I | Two-way ANOVA | Females = 5  Males = 5 | N/A | N/A |
| 5.J | Two-way ANOVA | Females = 4  Males = 5 | N/A | N/A |
| 5.K | Mixed-effects analysis  Inset: Two-way ANOVA | SAL = 4, PSI= 5 | Familiar Mouse: Treatment (F (1,7) = 9.056, p= 0.0197), Treatment x Mouse (F (3.647,25.43) = 3.221, p= 0.032)  Inset: F (1,14) = 9.169, p= 0.009 | Inset: Familiar Mouse: p= 0.0317 |
| 5.L | Two-way ANOVA | SAL = 4, PSI= 5 | N/A | N/A |
| 5.M | Mixed-effects analysis  Inset: Two-way ANOVA | SAL = 4, PSI= 3 | N/A | N/A |
| 5.N | Two-way ANOVA | SAL = 4, PSI= 3 | Mean Z-Score: Treatment (F (1,14) = 4.059, p= 0.0636),  Peak Z-Score: Treatment x Mouse interaction (F (1,14) = 5.131, p= 0.399) | Novel Mouse: SAL vs PSI: p= 0.0326  SAL: Familiar vs Novel Mouse: p= 0.0353 |

## **Statistics Table Supplementary Data 1.**

| Figure | Statistical test | | Group n | Main analysis result | | | Post-hoc multiple comparisons of interest | |
| --- | --- | --- | --- | --- | --- | --- | --- | --- |
| S1.A | One-way ANOVA | SAL+SAL = 9, MDL +SAL = 8, WAY+ SAL = 5, SAL+PSI = 9, MDL+PSI = 8, WAY+ PSI= 8 | | | All SAL-treated mice: F (2, 19) = 1.684, p= 0.0081  All PSI-treated mice: F (2,21) = 2.098, p< 0.0001 | | | SAL+SAL vs WAY+SAL: p= 0.0062, SAL+PSI vs MDL+PSI: p= 0.0029,  SAL+PSI vs WAY+ PSI: p< 0.0001 |
| S1.B | Two-way ANOVA | SAL+SAL = 9, MDL +SAL = 8, WAY+ SAL = 5, | | | Time (F (2.632, 49.53) = 4.398, p= 0.0106) | | | N/A |
| S1.C | Two-way ANOVA | SAL+PSI = 9, MDL+PSI = 8, WAY+ PSI= 8 | | | Time (F (4.204, 87.15) = 5.438, p= 0.0005);  Treatment (F (2,21) = 5.604), p= 0.0112) | | | SAL+PSI vs WAY+PSI: 25 min (p= 0.0228), 30 min (p= 0.0158), 35 min (p= 0.0275), 40 min (p= 0.0428),  SAL+PSI vs MDL+PSI: 50 min (p= 0.0419) |
| S1.D | Mixed-effects analysis | SAL+SAL = 9, MDL +SAL = 8, WAY+ SAL = 5, | | | Time (F (5.728, 119.4) = 3.327, p= 0.0052), Treatment (F (2.22) = 4.417, p= 0.0244) | | | SAL+SAL vs WAY+ SAL: 0 min (p= 0.0206), 5 min (p= 0.0398), 25 min (p= 0.0257), 65 min (p= 0.027), 70 min (p= 0.0112)  MDL +SAL vs WAY+ SAL: 45 min (p= 0.0258), 65 min (p= 0.0099) |
| S1.E | Mixed-effects analysis | SAL+PSI = 9, MDL+PSI = 8, WAY+ PSI= 8 | | | Time (F (3.531, 68.59) = 18.1, p< 0.0001), Time x treatment (F (52, 505) = 1.665, p= 0.0034 | | | SAL+PSI vs MDL+PSI: 0 min (p= 0.0398), 85 min (p= 0.0429)  SAL+PSI vs WAY+PSI: 75 min (p= 0.021),  MDL+PSI vs WAY+PSI: 75 min (p= 0.0007), 80 min (p= 0.02), 90 min (p= 0.0081), 95 min (p= 0.0319), 110 min (p= 0.025), 115 min (p= 0.0113), 120 min (p= 0.0152) |
| S1.F | One-way ANOVA | | SAL+SAL = 9, MDL+SAL = 8, WAY+SAL = 5, SAL+PSI = 9, MDL+PSI = 8, WAY+PSI = 7 | All SAL-treated mice: F (2,19) = 1.19, p= 0.0364  All PSI-treated mice: F (2,21) = 0.6772, p= 0.0366 | | | SAL+SAL vs MDL +SAL: p= 0.0339  SAL+PSI vs MDL+PSI: p= 0.0328 | |
| S1.G | One-way ANOVA | | SAL+SAL = 8, MDL+SAL = 8, WAY+SAL = 5, SAL+PSI = 9, MDL+PSI = 8, WAY+PSI = 7 | N/A | | | N/A | |
| S1.H | One-way ANOVA | SAL+SAL = 8, MDL +SAL = 8, WAY+SAL = 7, SAL+PSI = 9, MDL+PSI = 8, WAY+PSI= 5 | | | | All PSI-treated mice: (F (2,19) = 1.669, p= 0.0007) | | SAL+PSI vs WAY+ PSI: p= 0.0018, MDL+PSI vs WAY+ PSI: p= 0.0009 |
| S1.I | Two-way ANOVA | SAL+SAL = 8, MDL+SAL = 8, WAY+SAL = 7 | | | | Time (F (3.625,65.25)= 6.525, p= 0.0003), Time x treatment interaction (F (7.25,65.25)= 2.213, p= 0.0425) | | SAL+SAL vs WAY+ SAL: 0 min (p= 0.0399) |
| S1.J | Two-way ANOVA | SAL+PSI = 9, MDL+PSI = 8, WAY+PSI = 5 | | | | Time x treatment interaction (F (7.734,73.47) = 2.186, p= 0.0399 | | SAL+PSI vs WAY+ PSI: 45 min (p= 0.0496), 55 min (p= 0.0489) |
| S1.K | Mixed-effects analysis | SAL+SAL = 8, MDL+SAL = 8, WAY+SAL = 7 | | | | Time (F (5.062,96.96) = 16.36, p< 0.0001) | | N/A |
| S1.L | Mixed-effects analysis | SAL+PSI = 9, MDL+PSI = 8, WAY+PSI= 5 | | | | Time (F (4.341,81.64) = 13.79, p< 0.0001), Time x treatment interaction (F (52,489) = 1.496, p= 0.0172) | | SAL+PSI vs WAY+ PSI: 0 min (p= 0.0289),  SAL+PSI vs MDL+ PSI: 20 min (p= 0.0165) |
| S1.M | One-way ANOVA | | SAL+SAL = 8, MDL+SAL = 8, WAY+SAL = 7, SAL+PSI = 9, MDL+PSI = 8, WAY+PSI = 5 | N/A | | | N/A | |
| S1.N | One-way ANOVA | | SAL+SAL = 8, MDL+SAL = 8, WAY+SAL = 7, SAL+PSI = 6, MDL+PSI = 8, WAY+PSI = 4 | All SAL-treated mice: F (2,19) = 1.343, p= 0.0069 | | | SAL+SAL vs MDL +SAL: p=0.0366,  SAL+SAL vs WAY+ PSI: p= 0.0101 | |

## **Statistics Table Supplementary Data 2.**

| Figure | Statistical test | Group n | Main analysis result | Post-hoc multiple comparisons of interest |
| --- | --- | --- | --- | --- |
| S2.A | t-test | MDL+SAL= 8, MDL+PSI = 8 | N/A | N/A |
| S2.B | Two-way ANOVA | MDL+SAL= 8, MDL+PSI = 8 | Time (F (3.502,48.39) = 3.448, p= 0.0186), Time x treatment interaction (F (3.502,48.39) = 4.423, p< 0.0056) | N/A |
| S2.C | Mixed-effects analysis | MDL+SAL= 8, MDL+PSI = 8 | Time (F (3.002,36.71) = 6.813, p= 0.0009), Treatment (F (1.14) = 8.965, p= 0.0097), Time x treatment interaction (F (3.002,36.71) = 4.398, p= 0.0096) | 45 min (p= 0.0297) |
| S2.D | t-test | MDL+SAL = 5, MDL+PSI= 7 | N/A | N/A |
| S2.E | Two-way ANOVA | WAY+SAL= 5, WAY+PSI = 7 | N/A | N/A |
| S2.F | Mixed-effects analysis | WAY+SAL= 8, WAY+PSI = 8 | Time (F (2.577,33.2) = 6.192, p= 0.0028), Treatment (F (1.14) = 5.376, p= 0.0361), Time x treatment interaction (F (26,355) = 3.114, p< 0.0001) | 75 min (p= 0.0446), 90 min (p= 0.0478) |
| S2.G | t-test | MDL+SAL= 8, MDL+PSI = 8 | t (14) = 2.581, p= 0.0218 | N/A |
| S2.H | Two-way ANOVA | MDL+SAL= 8, MDL+PSI = 8 | Time (F (11,154) = 2.159, p= 0.0194) | N/A |
| S2.I | Mixed-effects analysis | MDL+SAL= 8, MDL+PSI = 8 | Time (F (3.01,36.12) = 5.715, p= 0.0026), Treatment (F (1,14) = 8.987, p= 0.0096), Time x treatment (F (26,312) = 1.602, p= 0.0342) | 60 min (p= 0.0365) |
| S2.J | t-test | MDL+SAL= 7, MDL+PSI = 5 | t (10) = 2.478, p= 0.0327 | N/A |
| S2.K | Two-way ANOVA | WAY+SAL = 5, WAY+PSI = 7 | Time (F (2.585,25.85) = 4.325, p= 0.0169), Treatment (F (1,10) = 7.749, p= 0.0193), Time x treatment (F (2.585,25.85) = 4.067, p= 0.0211) | N/A |
| S2.L | Mixed-effects analysis | WAY+SAL= 8, WAY+PSI = 8 | Time (F (3.559,43.53) = 18.03, p< 0.0001), Treatment (F (1,14) = 6.696, p= 0.0215) | N/A |

## **Statistics Table Supplementary Data 3**

| Figure | Statistical test | Group n | Main analysis result | Post-hoc multiple comparisons of interest |
| --- | --- | --- | --- | --- |
| S3.B | Two-way ANOVA | Females = 18  Males = 8 | N/A | N/A |
| S3.C | Two-way ANOVA | Females = 18  Males = 8 | N/A | N/A |
| S3.D | Two-way ANOVA | Females = 18  Males = 8 | N/A | N/A |
| S3.E | t-test | Females = 8  Males = 8 | N/A | N/A |
| S3.F | t-test | Females = 8  Males = 8 | t (13) = 2.381, p = 0.0333 | N/A |
| S3.G | Two-way ANOVA | SAL+SAL = 18  SAL+PSI = 16 | Time (F (11.02,341.9) = 20.85, p< 0.0001) | N/A |
| S3.H | t-test | SAL+SAL = 18  SAL+PSI = 16 | t (32) = 1.71, p= 0.097 | N/A |
| S3.I | t-test | SAL+SAL = 18  SAL+PSI = 16 | t (32) = 2.043, p= 0.0494 | N/A |
| S3.J | t-test | SAL+SAL = 8  SAL+PSI = 6 | t (8.016) = 2.383, p= 0.0443 | N/A |
| S3.K | t-test | SAL+SAL = 8  SAL+PSI = 6 | N/A | N/A |
| S3.L | Two-way ANOVA | SAL+SAL = 8  SAL+PSI = 7 | N/A | N/A |
| S3.M | Two-way ANOVA | SAL+SAL = 8  SAL+PSI = 7 | N/A | N/A |
| S3.N | Two-way ANOVA | SAL+SAL = 8  SAL+PSI = 7 | N/A | N/A |
| S3.O | t-test | SAL+SAL = 8  SAL+PSI = 7 | N/A | N/A |
| S3.P | t-test | SAL+SAL = 8  SAL+PSI = 7 | N/A | N/A |

## **Statistics Table Supplementary Data 4.**

| Figure | Statistical test | Group n | | Main analysis result | | Post-hoc multiple comparisons of interest |
| --- | --- | --- | --- | --- | --- | --- |
| S4.A | One-way ANOVA | SAL+SAL = 18, MDL+SAL = 8, WAY+SAL = 8, SAL+PSI = 16, MDL+PSI= 8, WAY+PSI= 8 | | All PSI-treated mice: F (2,29) = 1.868, p= 0.0107 | | SAL+PSI vs MDL+PSI: p= 0.0252  SAL+PSI vs WAY+PSI: p= 0.023 |
| S4.B | One-way ANOVA | SAL+SAL = 18, MDL+SAL = 8, WAY+SAL = 8, SAL+PSI = 16, MDL+PSI = 8, WAY+PSI= 8 | | All PSI-treated mice: F (2,29) = 0.5308, p= 0.0008 | | SAL+PSI vs MDL+PSI: p= 0.0007  SAL+PSI vs WAY+PSI: p= 0.0243 |
| S4.C | t-test | | SAL+SAL = 18  SAL+PSI = 16 | N/A | N/A | |
| S4.D | t-test | | SAL+SAL = 18  SAL+PSI = 16 | t (32) = 2.164, p= 0.0381 | N/A | |
| S4.E | t-test | | SAL+SAL = 8  SAL+PSI = 6 | t (10.14) = 2.208, p= 0.0514 | N/A | |
| S4.F | t-test | | SAL+SAL = 8  SAL+PSI = 6 | t (11.99) = 2.1, p= 0.0575 | N/A | |
| S4.G | Mixed-effects analysis | | SAL+SAL = 18, MDL+SAL = 8, WAY+ SAL = 8 | Time (F (11.85,348.9) = 17.17, p< 0.0001) | N/A | |
| S4.H | Mixed-effects analysis | | SAL+PSI = 16, MDL+PSI = 8, WAY+PSI = 8 | Time (F (10.05,286.9) = 18.04, p< 0.0001), Treatment (F (2.29) = 6.626, p= 0.0043) | MDL+PSI vs WAY+ PSI: 4 min (p= 0.0107), 6 min (p= 0.0465), 16 min (p= 0.0297), 17 min (p= 0.0278), 18 min (p= 0.0151), 19 min (p= 0.032), 20 min (p= 0.0068), 23 min (p= 0.0329)  SAL+PSI vs WAY+ PSI: 6 min (p= 0.0227), 11 min (p= 0.0188), 12 min (p= 0.0123), 13 min (p= 0.0256), 18 min (p= 0.0236), 21 min (p= 0.039), 23 min (p= 0.0006), 29 min (p= 0.0165) | |
| S4.I | One-way ANOVA | | SAL+SAL = 18, MDL+SAL = 8, WAY+ SAL = 8, SAL+PSI = 16, MDL+PSI = 8, WAY+PSI = 8 | F (5,60) = 3.001, p= 0.0176 | N/A | |
| S4.J | One-way ANOVA | | SAL+SAL = 18, MDL+SAL = 8, WAY+SAL = 8, SAL+PSI = 16, MDL+PSI = 8, WAY+PSI = 8 | N/A | N/A | |

## **Statistics Table Supplementary Data 5.**

| Figure | Statistical test | Group n | Main analysis result | Post-hoc multiple comparisons of interest |
| --- | --- | --- | --- | --- |
| S5.A | One-way ANOVA | SAL+SAL = 16, MDL+SAL = 8, WAY+SAL = 8, SAL+PSI = 14, MDL+PSI = 7, WAY+PSI= 7 | All SAL-treated mice: F (2,29) = 0.3241, p= 0.0287 | SAL+SAL vs MDL +SAL: p= 0.0168 |
| S5.B | One-way ANOVA | SAL+SAL = 16, MDL+SAL = 8, WAY+SAL = 8, SAL+PSI = 14, MDL+PSI = 7, WAY+PSI= 7 | All SAL-treated mice: F (2,29) = 2.159, p= 0.0141  SAL+PSI vs WAY+ PSI: t (11.17) = 2.108, p= 0.0584 | SAL+SAL vs MDL +SAL: p= 0.0077 |
| S5.C | Two-way ANOVA | SAL+SAL = 16, MDL+SAL = 8, WAY+SAL = 8, | Time: F (10.49,269.2) = 12.31, p< 0.0001 | N/A |
| S5.D | Two-way ANOVA | SAL+PSI = 14, MDL+PSI = 7, WAY+PSI= 7 | Time: F (10.56,306.4) = 7.905, p< 0.0001 | N/A |
| S5.E | One-way ANOVA | SAL+SAL = 16, MDL+SAL = 8, WAY+SAL = 8, SAL+PSI = 14, MDL+PSI = 7, WAY+PSI= 7 | N/A | N/A |
| S5.F | One-way ANOVA | SAL+SAL = 16, MDL+SAL = 8, WAY+SAL = 8, SAL+PSI = 14, MDL+PSI = 7, WAY+PSI= 7 | N/A | N/A |

## **Statistics Table Supplementary Data 6.**

| Figure | Statistical test | Group n | Main analysis result | Post-hoc multiple comparisons of interest |
| --- | --- | --- | --- | --- |
| S6.A | One-way ANOVA | SAL+SAL = 10, MDL+SAL = 9, WAY+SAL = 8, SAL+PSI = 8, MDL+PSI = 8, WAY+PSI= 7 | N/A | N/A |
| S6.B | One-way ANOVA | SAL+SAL = 10, MDL+SAL = 9, WAY+SAL = 7, SAL+PSI = 8, MDL+PSI= 8, WAY+PSI= 8 | All PSI-treated mice: F (5,44) = 6.315, p= 0.0108 | SAL+PSI vs MDL+PSI: p= 0.0694 |
| S6.C | Mixed effects analysis | SAL+SAL = 10, MDL+SAL = 9, WAY+SAL = 7, | Time: F (8.033,189) = 33.76, p< 0.0001 | N/A |
| S6.D | Mixed effects analysis | SAL+PSI = 8, MDL+PSI= 8, WAY+PSI= 8 | Time: F (5.316,113.9) = 35.55, p< 0.0001 | N/A |
| S6.E | One-way ANOVA | SAL+SAL = 10, MDL+SAL = 9, WAY+SAL = 7, SAL+PSI = 8, MDL+PSI= 8, WAY+PSI= 8 | N/A | N/A |
| S6.F | One-way ANOVA | SAL+SAL = 10, MDL+SAL = 9, WAY+SAL = 7, SAL+PSI = 8, MDL+PSI= 8, WAY+PSI= 8 | N/A | N/A |
